# Mixtures Biotransformation: Multilayer Molecular Networking of Kratom Liver Metabolites

**DOI:** 10.1021/acs.jnatprod.5c01235

**Published:** 2026-02-10

**Authors:** William J. Crandall, Jaclyn Weinberg, Ken Liu, Choon-Myung Lee, Grant Singer, Edward T. Morgan, Dean P. Jones, Cassandra L. Quave

**Affiliations:** † Molecular and Systems Pharmacology, 1371 Emory University, 201 Dowman Drive, Atlanta, Georgia 30322, United States; ‡ Department of Chemistry, Emory University, 201 Dowman Drive, Atlanta, Georgia 30322, United States; § Department of Medicine: Pulmonary, Allergy, Critical Care, and Sleep Medicine, Emory University, 201 Dowman Drive, Atlanta, Georgia 30322, United States; ∥ Department of Pharmacology & Chemical Biology Professor Emeritus, Emory University, 201 Dowman Drive, Atlanta, Georgia 30322, United States; ⊥ Department of Dermatology, Emory University, 201 Dowman Drive, Atlanta, Georgia 30322, United States; # Center for the Study of Human Health, Emory University, 201 Dowman Drive, Atlanta, Georgia 30322, United States; ∇ Emory University Herbarium, Emory University, 1462 Clifton Rd NE, Room 102, Atlanta, Georgia 30322, United States

## Abstract

Metabolites generated from therapeutic drugs can exhibit diverse pharmacological effects, making knowledge of metabolites important to understand the overall mechanisms of therapeutic action. Methods to study the metabolism of single or simple mixtures of bioactive agents are often insufficient to address the chemical diversity of natural product mixtures. Molecular network analysis has emerged as a powerful approach to gain an understanding of complex chemical mixtures. In the present study, we used *in vitro* biotransformation and molecular network analysis of single compounds and extracts of the medicinal plant, kratom (*Mitragyna speciosa* Korth., Rubiaceae), to test the utility for characterizing natural product mixtures. We analyzed biotransformation products from single compounds and multiple chemotypes of kratom leaf extracts using human liver S9 and liquid chromatography–high-resolution mass spectrometry. Multilayer molecular networking of the polymetabolism of the entire natural product mixture provided predicted metabolites from precursors in a semitargeted manner. Both phase I and phase II metabolites of precursors were detected, with the metabolic profiles of individual components being substantially different in the context of total extracts. Application of molecular networking to mixture metabolism will enhance metabolite identification and the understanding of the therapeutic and toxicological mode(s) of action of pharmacologically active plants.

## Graphical abstract

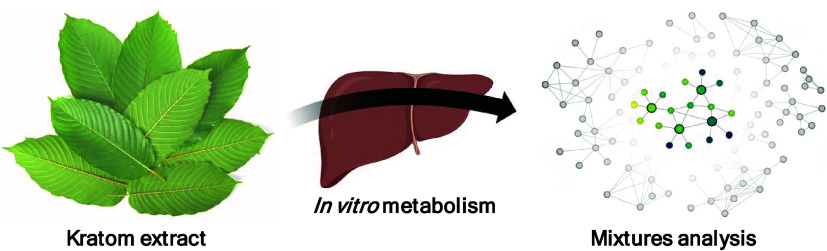

Kratom (*Mitragyna speciosa* Korth.) is a plant from the Rubiaceae (coffee) family native to various regions of Southeast Asia. First described by Dutch botanist Pieter Willem Korthals, the finalized name prefix “Mitra” was likely given as the stigma were described as “mitraeforme”, Latin for miter-shaped, changed from the originally named, *Stephengyne*.^1^ Kratom has medicinal uses for its stimulant-like, pain-relieving, stress-reducing, antidiarrheal, and antipyretic effects.^2–6^ Typically, kratom is consumed by chewing the leaves or creating a tea using dried or fresh leaves in water.^2,3^ In the United States, formulations such as extracts, pills, capsules, and suspensions are sold.^5^ Its first documented use for the treatment of pain dates back to 1897, where it was described as a substitute for opium.^7^ This activity is, in part, due to the presence of the main indole alkaloid present in the leaves of the plant, mitragynine (MG). In addition to opioid receptor affinities, other indole alkaloids within kratom have shown affinity for adrenergic and serotonin receptors,^8,9^ further complicating the understanding of the overall effect of the plant. *M. speciosa* is distinguished from 9 other species within the genus by containing detectable quantities of mitragynine and 7-hydroxymitragynine (7OH-MG).^2,3^ There are 54 known indole and oxindole alkaloids in *M. speciosa*,^10^ which vary greatly depending on the age and the eco-/chemotype, with the relative amount of mitragynine contributing anywhere from 12 to 66% of the total alkaloidal content of the leaves.^2^ Various formulations of *M. speciosa* extracts and specific metabolites (e.g., 7-hydroxymitragynine) have flooded US markets and are sold in vape shops, gas stations, and bodegas, necessitating better means to evaluate the authenticity and content of medicinally active components. Furthermore, kratom does not currently meet the regulatory criteria of the DSHEA Act to be considered a dietary supplement and thus falls into a regulatory gray zone. Improved understanding of the alkaloids and interconversions to bioactive compounds is needed to ensure safety and, if appropriate, inform regulatory criteria.

Mitragynine, first isolated in 1921,^11^ is a partial agonist of the μ-opiate receptor (MOR) with a relatively low potency compared to the prototypical opioid agonist, morphine.^12–15^ Comparatively, 7-hydroxymitragynine is reported to be about 13 times more potent than morphine, and 46 times more than mitragynine.^12,13^ 7-Hydroxymitragynine is a major metabolite of mitragynine, formed largely by cytochrome P450 (CYP), 3A4.^16,17^ While its abundance is relatively low in the plant leaves, its formation during hepatic first-pass metabolism suggests it plays a role in the overall efficacy of kratom; however, the extent of this contribution may be overestimated.^18,19^ Mitragynine pseudoindoxyl is another oxidative metabolite of mitragynine and exhibits partial agonism of MOR with a measured *in vitro* potency of ∼120-fold and ∼31-fold greater than mitragynine and 7-hydroxymitragynine, respectively.^15,20^ Mitragynine pseudoindoxyl forms both in human plasma^21^ and through a CYP3A4-mediated pathway,^16^ although in both cases, enzymatic inhibition did not fully inhibit metabolite formation, indicating potential nonenzymatic mechanisms of oxidative rearrangement. The stability of mitragynine pseudoindoxyl is low, where intramolecular 1,2-semipinacol rearrangement occurs in 20 min in a protic environment producing three observed stereoisomers.^22^ Subcutaneous injection of each individual diastereomer did not provide significantly different ED_50_’s, and thus the effect is thought to be mediated by the equilibrated mixture of all three.^22^ Major demethylated metabolites 9-*O*-demethylmitragynine (9-hydroxycorynantheidine) and 16-carboxymitragynine were found to have lower potencies than mitragynine, with 16-carboymitragynine exhibiting no agonism up to 100 μM at hMOR.^19^ Further transformation yields sulfated and glucuronidated metabolites, of which glucuronyl-9-hydroxycorynantheidine has been shown to have weak *in vitro* affinity for mMOR.^16,23^

While mitragynine typically comprises most of the alkaloidal content in the kratom leaf (12–66%), other compounds can comprise significant portions.^6,10,14,24^ Paynantheine, a C20 epimer ethenyl analog of mitragynine, has shown moderate affinity for 5-HT_1A_ and 5-HT_2B_ subtypes.^8^ Corynoxeine has been shown to inhibit vascular smooth muscle cell proliferation,^25^ and its C20 ethyl isomers corynoxine and corynoxine B have been studied for their ability to induce autophagy in neuronal cells.^26,27^ Speciofoline, abundant in chemotypes of commercially available kratom,^14^ exhibits weak potency at hMOR, along with its stereoisomer isospeciofoline.^14,28^ While the metabolism and pharmacology of multiple kratom alkaloids and their related metabolites have been studied, many are undercharacterized, particularly in the context of their mixture.

The chemical complexity of related alkaloids and their conversion to activated metabolites present a large challenge in understanding the pharmacological effect of the entire plant. Due to the abundance of similarly related structures, liquid chromatography coupled to high-resolution tandem mass spectrometry (LC-HRMS/MS)-based molecular networking serves as the most direct method for simultaneously evaluating the transformative fates of these metabolites in the context of a complex mixture. The alkaloids of kratom are substrates for CYPs and, therefore, competition is expected. Competitive, noncompetitive, and time-dependent mechanisms of inhibition have been observed.^29,30^ In the case of mitragynine, it is primarily oxidized by CYP3A4 ^19^ and is a potent inhibitor of CYP2D6.^14,29–31^ While previous studies have investigated one chemical at a time, the potential for enzymatic inhibition, hetero- or homotropic cooperativity, and/or differing kinetics in the context of an alkaloid mixture necessitates investigation of kratom as a whole plant. In addition to examining the biotransformation profiles of each compound in a kratom extract, analysis of the mixture is likely to enhance the metabolite identification workflow. Utilizing knowledge-guided and orthogonal experimental criteria for metabolite annotation provides a streamlined and higher confidence method for metabolite ID.^32,33^ Here, we combined mass spectral data from *in vitro* human liver S9 biotransformation of kratom extracts in the form of MS^1^ and MS^2^ criteria, and knowledge from *in silico* biotransformation to build annotated molecular networks for metabolite ID in kratom.

## Results and Discussion

### Multilayer Molecular Networking Enables the Discovery of Metabolites Directly from Extracts

Although more concentrated extracts and purified forms of kratom are seen in the market, the majority of kratom is consumed in its entirety, by whole leaf.^34^ Therefore, the metabolic profile of each constituent can be affected by the other. Combining knowledge-based criteria such as known biotransformation of drugs in human systems, with both MS^1^ and MS^2^ experimental criteria enabled multiple levels of annotation for building precursor–metabolite molecular networks (Figure 1). Initially, a precursor was identified as having a technical triplicate detection and MS^2^ data in the kratom extract. Metabolites then required MS^2^ spectral similarity or both a *p* value <0.05 and a predicted mass using Biotransformer.^35^ A significant correlation with time was the final criterion. A piecewise slope was used for additional annotation to determine metabolite patterns over the incubation time points. Lastly, mass defect filtering was included in annotating features that had no predicted mass using Biotransformer 3.0. This is highlighted in the case of speciofoline incubation, where the network adding mass defect filtering increased total annotated metabolites from 15 to 34 (Figure S1). Final networks facilitated rapid annotation and discovery of precursor and metabolite relationships, which was crucial when examining the biotransformation products of kratom extracts. Importantly, for these classes of compounds, the final networks could be compared to nonenzymatic-based controls, indicating that not all metabolites detected are due to enzymatic biotransformation. Detection of some metabolites could be partially due to chemical degradation from instability of the precursor, as observed with the stereoisomerization of mitragynine pseudoindoxyl.^22^

**Figure 1:**
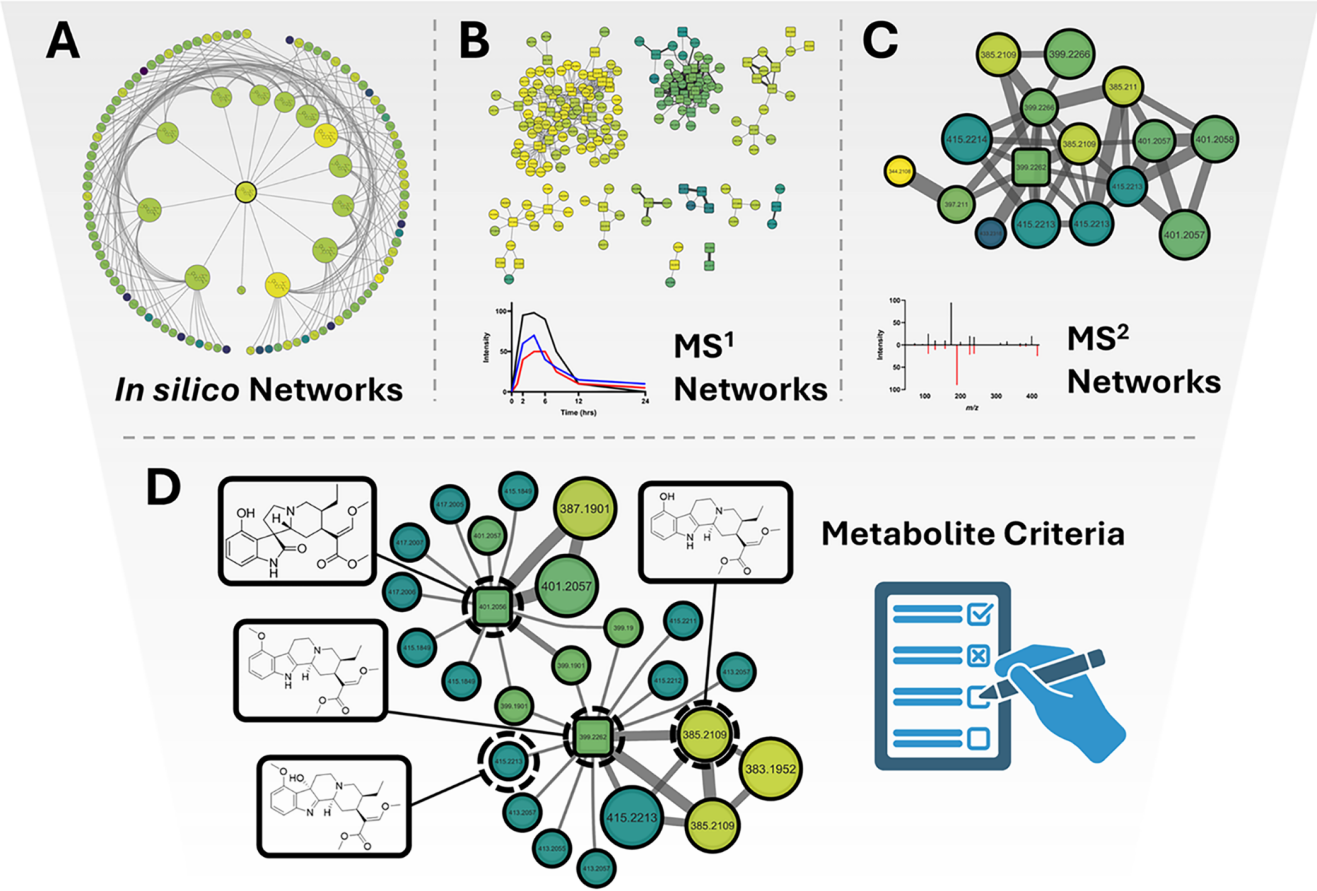
Multilayer molecular networking (MLMN) for metabolite identification in mixtures metabolism. (A) *In silico* biotransformation product predictions from Biotransformer 3.0 are combined with (B) peak area statistics over time course, and (C) spectral similarity networks generated from MS^2^ data. (D) The resulting precursor–metabolite network encompasses multiple annotation criteria enabling a streamlined approach to confident metabolite identification in a mixture setting.

Initial analysis by hydrophilic interaction liquid chromatography (HILIC) yielded seven annotated metabolites for mitragynine and paynantheine and nine metabolites for corynoxeine (Figure 2A). Incubation of the kratom extract yielded multiple complex networks (Figure 2B); however, isolating the known standards and creating a single-hop network allows for simpler interpretation (Figure 2C). Using the metabolites formed from single-compound incubation as reference standards, matching the *m*/*z* and retention time in the annotated extract network allowed for confirmation of a couple of metabolites from each compound. The list of metabolites can be found in Supporting Table S1. This showed that an unreported phase II metabolite (*m*/*z* 591.2554) of mitragynine was detected in both single-compound incubation (Figure 2A) and directly from the kratom mixture (Figure 2C). To the best of our knowledge, the *m*/*z* 591 has not been reported for mitragynine; however, it has been reported as a metabolite of the diastereomer, speciocilatine.^36^

**Figure 2:**
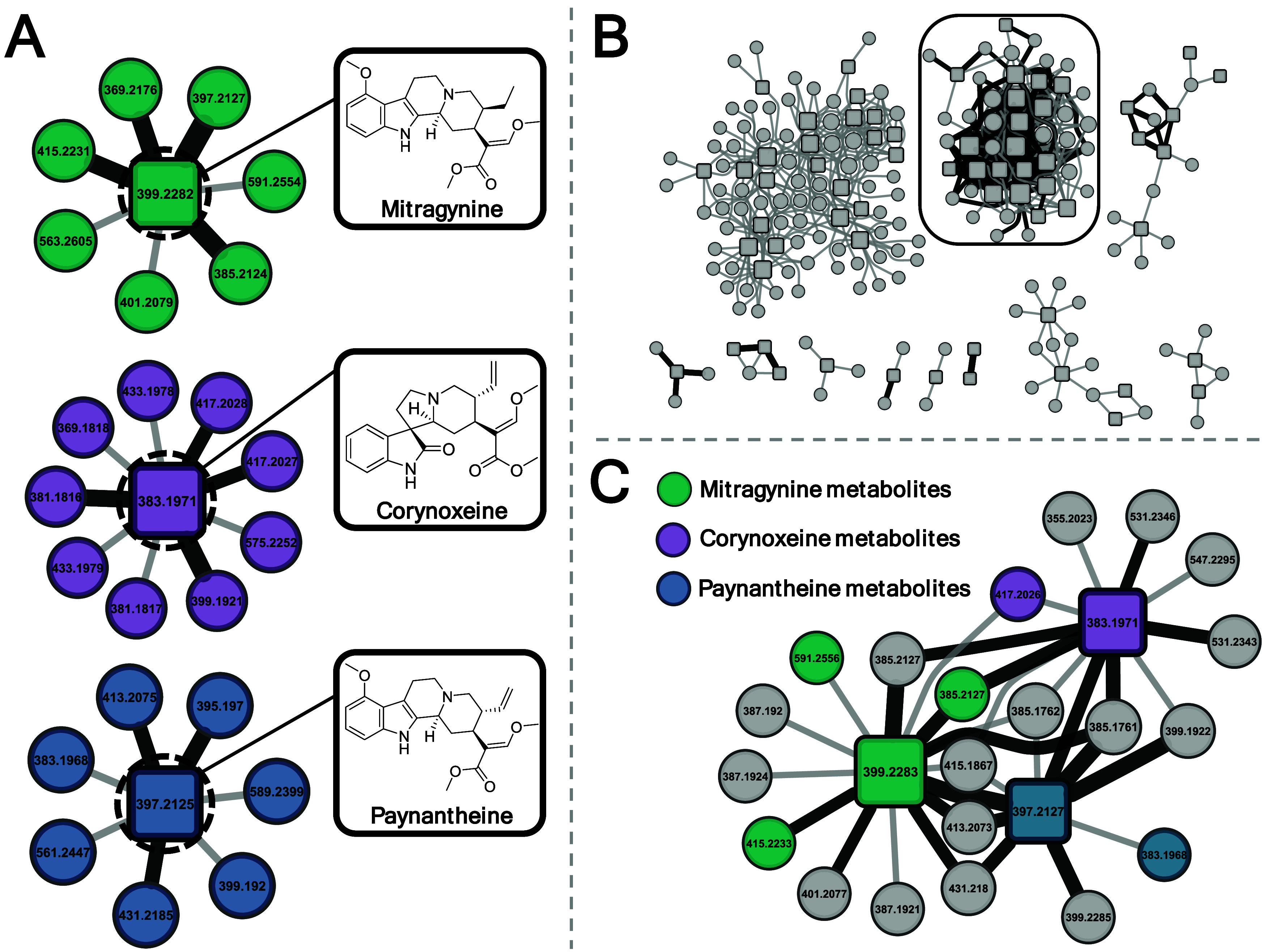
Reference network generation using authentic standards. (A) Single-compound networks from chemical standards (color-coded) analyzed using HILIC chromatography (LC-Orbitrap/MS) resulted in multiple phase I and II metabolites annotated. Metabolite nodes are annotated in Table S1. (B) Biotransformation of the entire kratom extract, KTP, yielded multiple large precursor–metabolite networks, with the network containing authentic standards circled. (C) Nearest neighbor network of the three chemical standards detected in the extract biotransformation. Colored nodes correspond to matching *m*/*z* and retention times of metabolites annotated based upon pure compound incubation in panel (A).

To further validate that the metabolite is in part formed from mitragynine, since there are three other diastereomers present in the extracts, a direct comparison of the phase II metabolite detection in extract K52 (Figure 3A), to that of an isotopically labeled mitragynine-d_3_ spiked K52 extract (Figure 3B) was performed. Since 7OH-MG is an abundantly formed mono-oxidative metabolite of mitragynine, the predicted structure is likely 7OH-MG-glucuronide (7OH-MG-G). This is in accordance with the MS^2^ spectrum, where the primary product ion is *m*/*z* 415.2222 (Figure 3C). Furthermore, MS^3^ fragmentation of the *m*/*z* 415 product ion matches the MS^2^ spectrum of a 7OH-MG standard. Unsurprisingly, Biotransformer 3.0 did not predict this metabolite since it also failed to predict 7OH-MG. However, a secondary prediction for the *m*/*z* 591 metabolite is the M+ ion of an N-oxidation-glucuronidation. N-oxide-mitragynine has been found as a constituent of kratom;^10^ however, fragmentation of the *m*/*z* 415 product ion shows an MS^3^ product ion at *m*/*z* 190, corresponding to an oxidation of the indole rings of the molecule, observed in 7OH-MG and not mitragynine-*n*-oxide. This is corroborated with the MS*^n^* data collected on the isotopically labeled *m*/*z* 594 metabolite, where the deuterium atoms are on the C9 methoxy, which also showed a *m*/*z* 193 product ion in the MS^3^ spectrum (Figure S2). It should be noted, however, that hydroxylation on the benzene indole ring is hard to distinguish from C7 hydroxylation by MS*^n^* alone. The relative intensity of *m*/*z* 397 was higher in the 7OH-MG standard MS^2^ spectra compared to that in the MS3^3^ spectra, indicating a different hydroxylation site (Figure 3F). Therefore, a purchased standard of 7OH-MG was incubated in the presence and absence of UDPGA (uridine 5′-diphosphoglucuronic acid), the only cofactor. Analysis of this incubation extract on C18 chromatography revealed two peaks, compared to HILIC analysis. The first peak was confirmed to be 7OH-MG-glucuronide (Figure 3D,E). The second glucuronide is therefore the result of hydroxylation on the indole benzene with subsequent glucuronidation (OH-MG-G).

**Figure 3:**
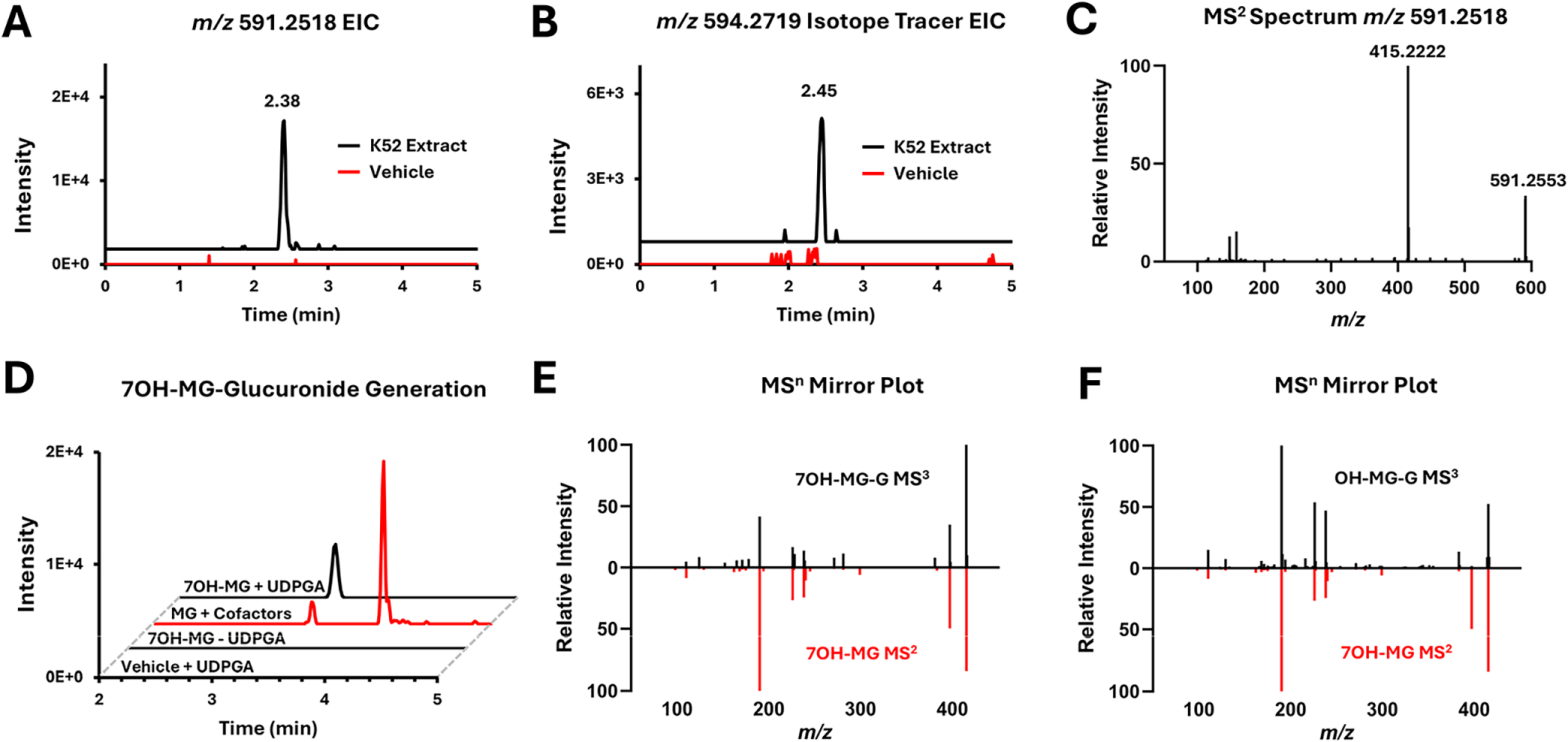
Putative identification of mitragynine phase II metabolites. (A) An unreported phase II metabolite (*m*/*z* 591.2518) of mitragynine was detected after 6 and 24 h incubation of kratom extract K52, with traces of the 24 h time point shown (LC-Q-Orbitrap/MS). (B) Isotopically labeled mitragynine incubated with the K52 extract in liver S9 fraction resulted in the detection of the calculated *m*/*z* at the expected retention time (LC-Q-Orbitrap/MS). (C) MS^2^ fragmentation of *m*/*z* 591.2518 shows a loss of glucuronide (−176 Da) and a single dominant product ion (*m*/*z* 415.222) matching the calculated mass of hydroxylated mitragynine (LC-Q-Orbitrap/MS). (D) Incubation of 7OH-MG with UDPGA cofactor in S9 shows the formation of the metabolite at a retention time of 3.40 min (7OH-MG + UDPGA trace), with no metabolite formed in the absence of UDPGA cofactor (7OH-MG – UDPGA trace) or in the absence of substrate (vehicle + UDPGA trace). Mitragynine incubated with all cofactors in S9 fractions (red trace) shows two peaks (3.39 min, 4.04 min) (LC-Q-Orbitrap/MS). (E) MS^3^ spectra of the *m*/*z* 415 product ion of the 7OH-MG-G metabolite (3.40 min) mirrored over that of a 7OH-MG standard (LC-Q-ITMS/MS). (F) MS^3^ spectra of *m*/*z* 415 product ion the OH-MG-G metabolite (4.04 min) mirrored over that of a 7OH-MG standard (LC-Q-ITMS/MS).

### Kratom Extract Chemotype Effects Biotransformation Profile

The three water decoction extracts of kratom leaves used in this study show distinct chemotypes, as depicted by PCA (Figure 4A). Extract K49 is distinguished by mitragynine and related diastereomers in the loading plot, which is highlighted in purple (Figure 4B). Extracts K52 and KTP were more complex by the analysis of the base peak chromatogram (Figure S3), with K52 containing the greatest relative abundances of Speciofoline and related isomers of *m*/*z* 401, highlighted in blue (Figure 4B). Extract KTP contained the greatest amount of corynoxine B and related *m*/*z* 385 isomers, which was putatively identified through MS^2^ database matching (Figure 4B). The three extract chemotypes can be crudely characterized by their relative abundances of these three main alkaloidal profiles, with K49 showing the greatest relative abundance of mitragynine (Figure S4).

**Figure 4:**
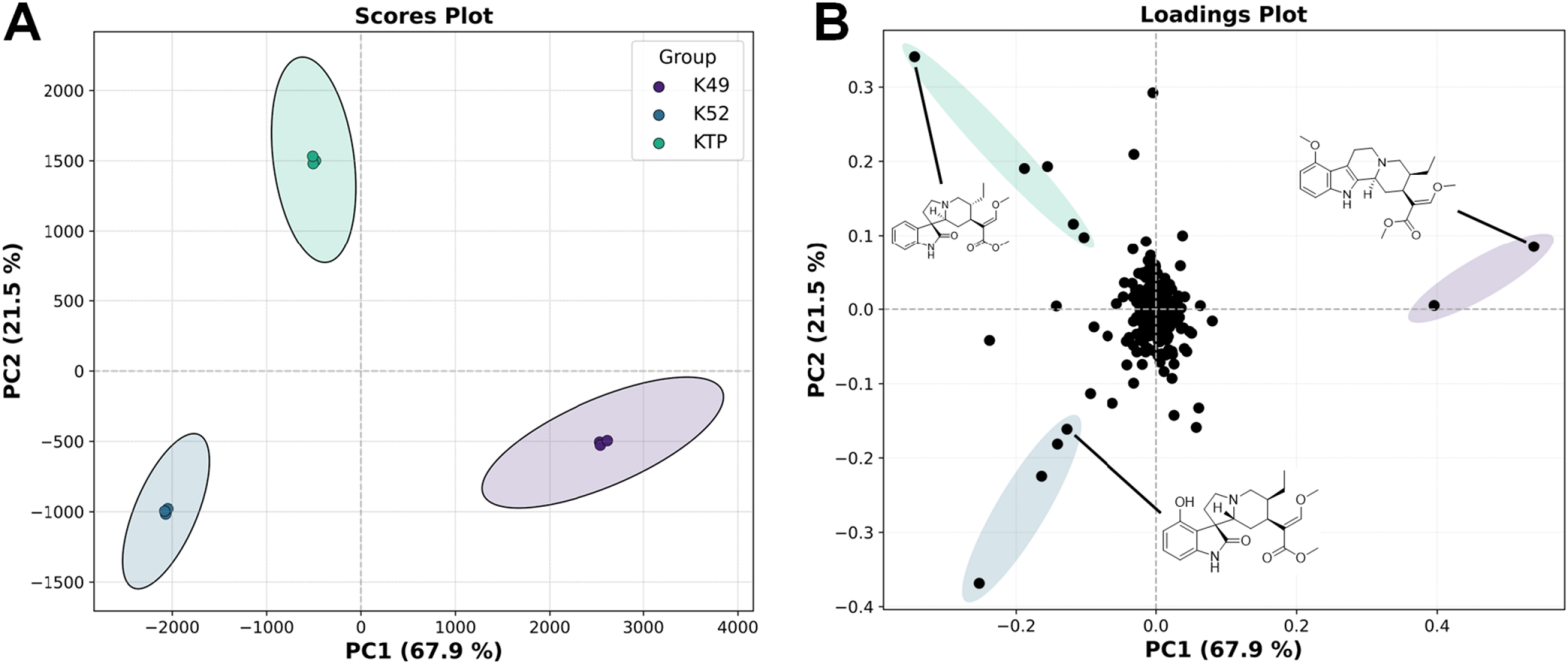
Principal component analysis (PCA) of Kratom extract chemotypes. (A) PCA scores plot of kratom extracts showing distinct chemotypes with ellipses showing hoteling T squared 95% confidence intervals. (B) Loadings plot showing three distinct *m*/*z* groups (highlighted by ellipses), which contribute most to the variance between groups. Mitragynine and *m*/*z* 399 diastereomer in purple, speciofoline and *m*/*z* 401 ions in blue, and corynoxine B (MS^2^ library matched) and related *m*/*z* 385 ions in green ellipses.

Different metabolic profiles for each chemotype of kratom are expected due to varying concentrations of precursors, differences in rates of enzymatic efficiencies, and inhibition by co-occurring precursors. This was observed when matched metabolites of pure mitragynine incubation were compared across the three extracts. Network analysis quickly showed visual differences in the annotated metabolites. The incubation of K49 resulted in 7 matched nodes, whereas the more chemically complex extracts K52 and KTP matched only 5 metabolites (Figure 5A). Similarly, additional metabolites of speciofoline were detectable in the K52 extract, compared to the KTP extract. In K49, speciofoline metabolites were undetectable, due to the low abundance of precursor in the extract (Figure S5). Across time points, each extract showed significantly different levels of 7OH-MG, after subtracting for 7OH-MG already present in the extract measured at the 0 h time point (Figure 5B,C). 7OH-MG formation decreases from 6 to 24 h time points (Figure 5B) while formation of the phase II, OH-mitragynine-glucuronide, metabolite increases from 6 to 24 h time points (Figure S7). Understanding the competitive formation of active metabolites such as 7-hydroxymitragynine in the context of a mixture is a critical factor in understanding the overall pharmacology of kratom, as there are numerous commercially available chemotypes of the plant.^14^

**Figure 5:**
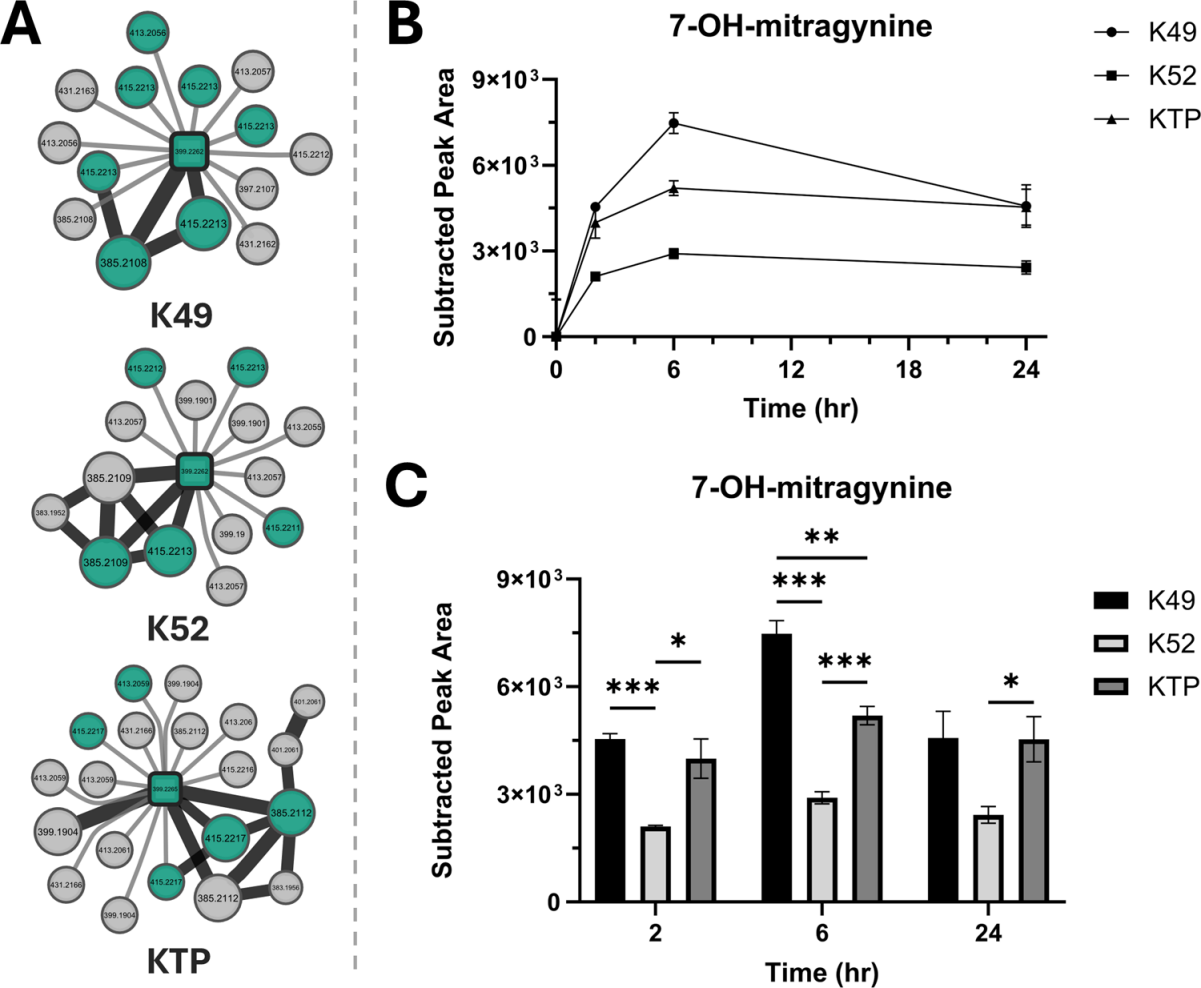
Chemotype influences 7-hydroxymitragynine formation. (A) Mitragynine-metabolite networks from incubation of each kratom extract type extract. Two additional phase I metabolites met the annotation criteria for K49, compared to K52 and KTP. (B) Relative abundance of 7OH-MG in each kratom extract at 0, 2, 6, and 24 h. Peak areas were subtracted from average baseline (0 h) levels in each extract (LC-Orbitrap/MS). (C) Statistical comparisons of time course data between treatment groups analyzed by two-way repeated measures ANOVA with Tukey’s post hoc analysis (*** = *p* < 0.001, ** = *p* < 0.01, * = *p* ≤ 0.05, *n* = 3 for each time point). 7OH-MG was confirmed with a purchased standard (Figure S6).

### Deconvolution of Kratom Metabolites

For mitragynine incubation, analysis by HILIC chromatography yielded better detection of phase II metabolites but was not adequate in the case of kratom, due to the numerous structurally similar alkaloids. Common phase I metabolites, 9-*O*-demethylmitragynine and 16-carboxymitragynine at *m*/*z* 385.2124, were predicted in a single node, with convoluted MS^2^ spectra (Figure 6A). Since the demethylation sites are on distinct sides of the molecule, MS^2^ fragmentation yields three distinct product ions: *m*/*z* 160, 226, and 238 for 9-*O*-demethylmitragynine (Figure S8), and *m*/*z* 174, 212, and 224 for 16-carboxymitragynine (Figure S9). An optimized gradient was developed for C18 chromatographic conditions, in which a greater number of phase I metabolites of mitragynine could be detected with two distinct peaks for both 9-*O*-demethylmitragynine and 16-carboxymitragynine (Figure 6B). While comparison to published MS^2^ spectra could be used to identify both forms, chromatographic separation is crucial for unknown identification. High-throughput chromatographic conditions are not adequate to profile the metabolic profiles of natural product mixtures such as this, where 10 mono-oxidative metabolites of mitragynine could be detected (*m*/*z* 415) that meet annotation criteria. A unique advantage of *in vitro* metabolism experiments is the ability to create nonenzymatic-based control conditions. Out of the 10 mono-oxidative metabolites, three were formed nonenzymatically in incubations with heat-inactivated S9 fractions (Figure 6C), indicating precursor instability.

**Figure 6:**
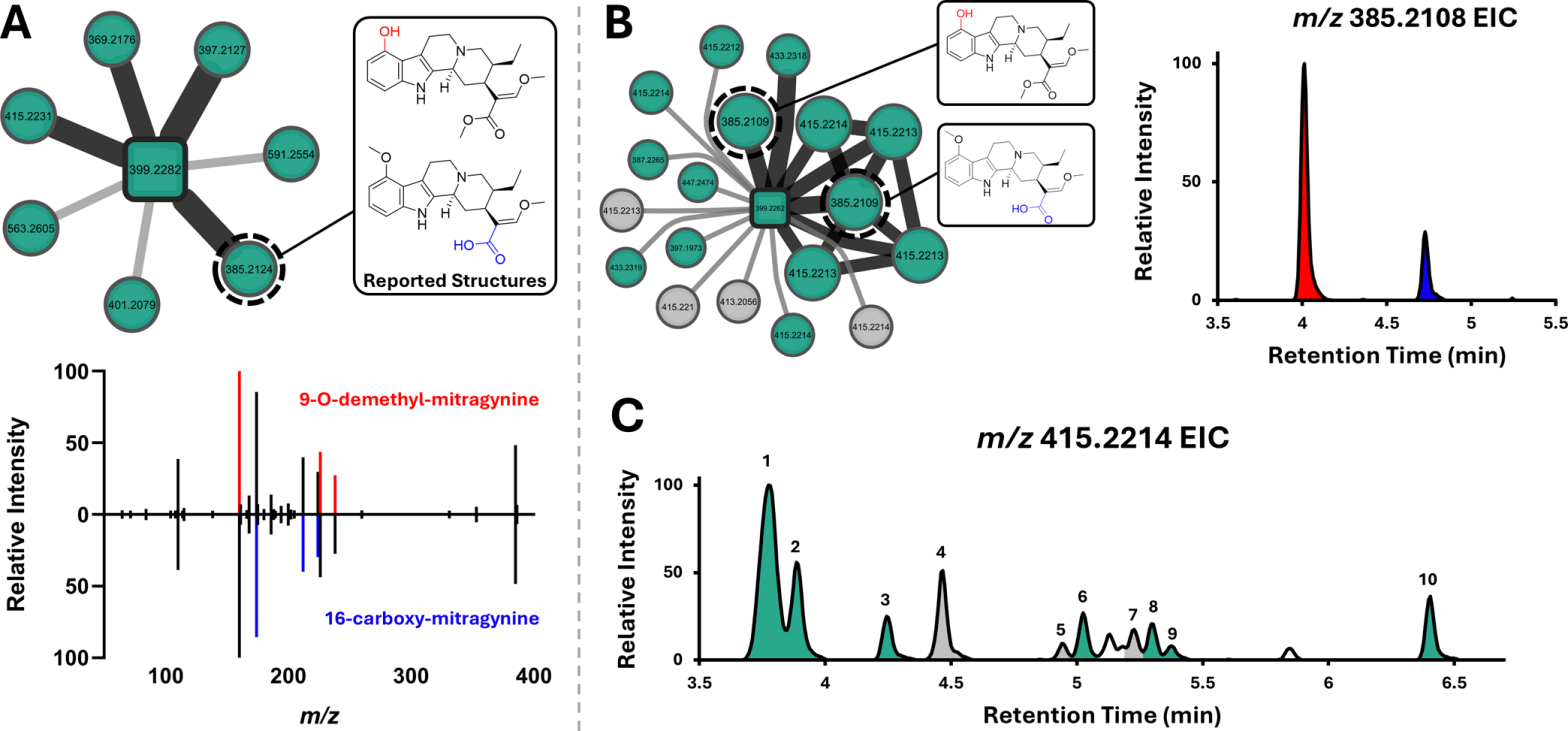
Metabolite Deconvolution. (A) Mitragynine standard (square node) incubation analyzed by HILIC chromatography yielded seven metabolites, two of which were phase II. Convoluted MS^2^ spectra for known metabolites 9-*O*-demethyl- and 16-carboxy-mitragynine could be resolved through comparison to the literature. (B) Mitragynine standard (square node) incubation analyzed by C18 chromatography yielded 14 detected phase I metabolites and successfully resolved the metabolites chromatographically. Metabolite nodes in the network that are gray were found to form nonenzymatically in heat-inactivated S9 controls. (C) In total, 10 mono-oxidative metabolites met annotation criteria, with 3 of the 10 (grayed peaks) being those that form nonenzymatically.

A list of mitragynine and speciofoline metabolites annotated by C18 chromatographic analysis can be found in a Supporting Information (Table S2). Metabolites detected in the mixture can be seen as combined networks for both K52 and KTP extracts, which were the only two chemotypes with appreciable abundances of speciofoline (Figure S7).

### Stereoisomerization of Kratom Alkaloids and Metabolites

The instability of 7-hydroxymitragynine leads to the 1,2-semipinacol rearrangement to the more potent mitragynine pseudoindoxyl^21^ of the same *m*/*z* 415. In human plasma, this is only partially blocked through the addition of a cocktail of protease inhibitors.^21^ Likewise, CYP3A4-catalyzed formation of mitragynine pseudoindoxyl is only partially inhibited by ketoconazole.^16^ Furthermore, in physiologically mimicked protic environments, mitragynine pseudoindoxyl is interconverted between three diastereomeric forms,^22^ void of any enzymatic system. This greatly complicates the landscape of kratom metabolism, where nonenzymatic-based stereoisomerism can quickly conflate the number of detectable metabolites and play an important role in pharmacological mechanisms of action. While this has been studied for the main alkaloid mitragynine regarding its action at the opiate receptors, there are numerous other metabolites that can undergo the same processes. Foremost are the three other diastereomers of mitragynine, which have been previously isolated and can be separated by reversed-phase C18 chromatography (Figure S10).^10^ As previously discussed by Angyal et al., stereoisomerism results in epimers at the spiro carbon and the C3 carbon. Energetic simulations of the four possible stereo configurations explained why only three stereoisomers were observed.^22^ Speciociliatine, the C3 epimer of mitragynine follows largely the same metabolic pathway as mitragynine.^36^ Therefore, if speciociliatine were metabolized to the pseudoindoxyl, stereoisomerization could result in identical pseudoindoxyl metabolites to that of mitragynine (Figure S11). Metabolism and stereoisomerization of speciogynine, the C20 epimer of mitragynine, could then result in three unique pseudoindoxyl metabolites if the energetics of this ethyl epimer remain similar. Convergent mechanisms of mitraciliatine, the C3 epimer of speciogynine, are therefore theoretically possible. This would result in 10 mono-oxidative metabolites with identical mass (415.2155 Da, C_23_H_30_N_2_O_5_) from just four diastereomeric precursors present in kratom (detailed in Figure S11).

Interconvertible stereoisomerization occurs not only for spiro – pseudoindoxyls, but also for spiro oxindoles,^37–41^ with significant pharmacological implications.^40^ Stereoisomerization has been observed with rhyncophylline and mitraphylline,^42^ two spiro oxindoles found in kratom.^10^ There are four known diastereomers of speciofoline found within kratom which differ at carbons 3 and 7 (Scheme 1).^10^ Upon examination of the nonenzymatic transformation products of speciofoline, conducted by heat inactivating the S9 fraction, four separate peaks of the same accurate mass were detected (Figure 7A). These also align with the peaks observed in the kratom extract itself (Figure S12). Regarding the conflation of metabolite ID, multiple demethylated metabolites of speciofoline were annotated (Table S2). Since speciofoline exhibits a hydroxyl on the indole ring, the only other observed demethylation sites are on methoxy acrylate moiety, of which hydrolysis of the ester to carboxylic acid is likely favored.^23^ This reaction is likely catalyzed by carboxylesterase 1 (CES1), which was shown to be inhibited by mitragynine and other select alkaloids from kratom in a concentration-dependent manner.^43^ Observed after 2 h incubation is a single peak, annotated as 16-carboxyspeciofoline (Figure 7B). However, the appearance of additional peaks of the same monoisotopic mass over time course further corroborates stereoisomerization of the structure, therefore producing four 16-carboxy metabolites (Figure 7B). A biotransformation pathway for speciofoline is proposed (Figure S13). Similarly annotated pathways for mitragynine, paynantheine, and corynoxeine have been found in the literature.^23,44,45^

**Figure 7:**
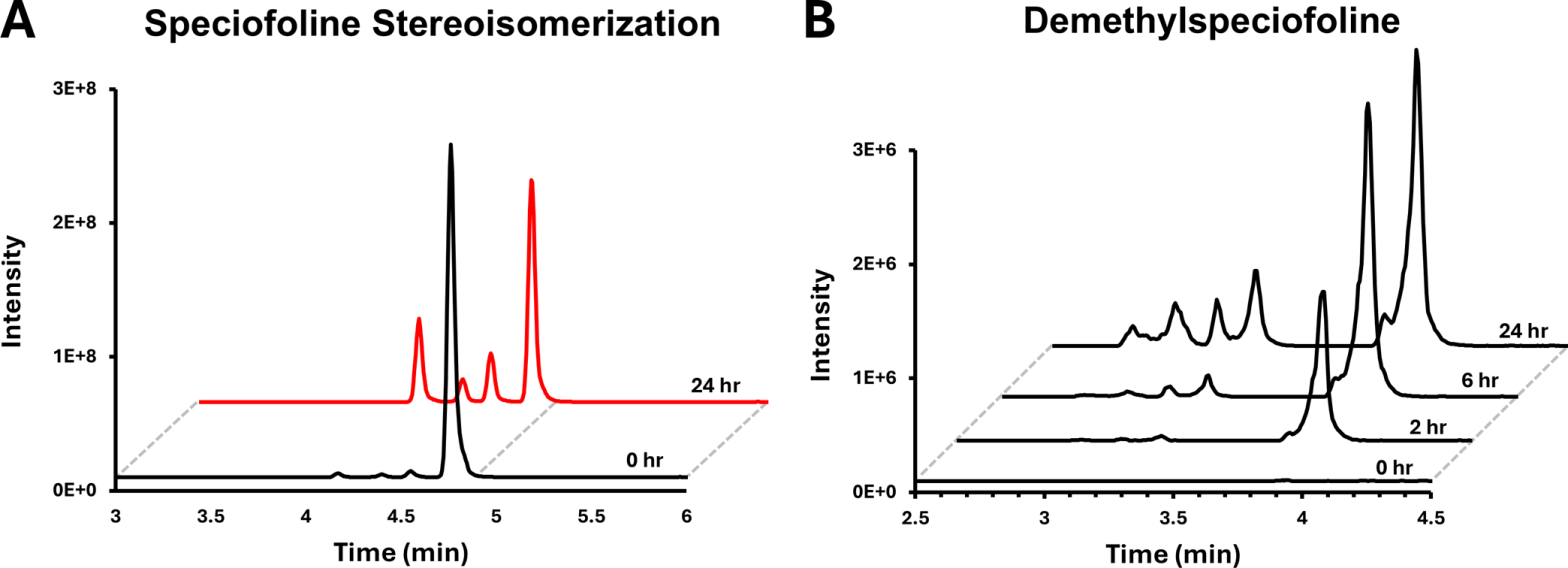
Stereoisomerization of speciofoline and demethylated metabolites. (A) Speciofoline incubated under heat-inactivated S9 conditions over 24 h. (B) Demethylated speciofoline metabolites (*m*/*z* 387.1900) over a time course under standard S9 reaction conditions.

**Scheme 1:**
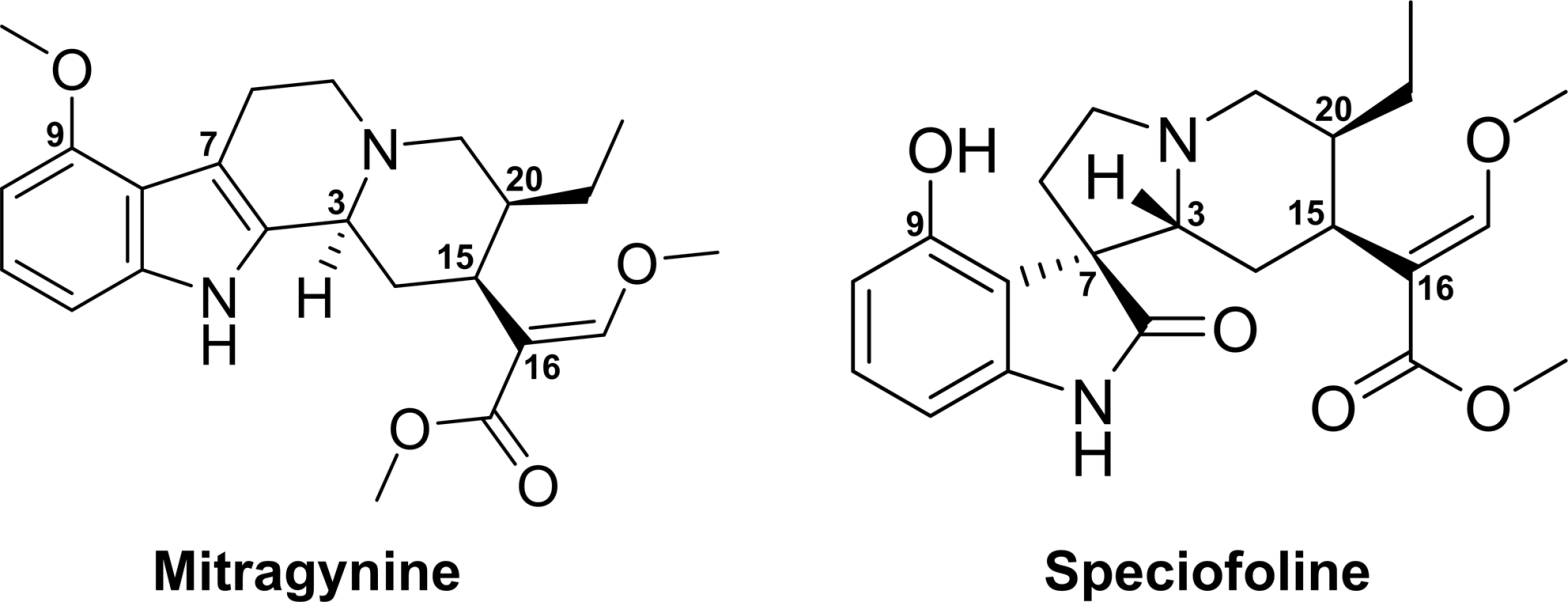
Kratom Alkaloids Mitragynine and Speciofoline

Speciofoline exhibits no measurable binding affinity at any opiate receptor subtype; however, isopseciofoline shows high nM affinity for MOR, with the other stereoisomers not yet tested.^14,28^ The indole dehydroxylated C20 stereoisomer analogs rhynchophylline and isorhynchophylline have been found to have vasodilatory effects through action on calcium channels,^46^ and mitraphylline antiproliferative activity.^47^ Although the half-life of speciofoline or similar stereoisomers has not been reported in humans, given that the mean half-life of mitragynine for the oral dose of kratom leaf material is about 1 day, it is reasonable to expect some stereoisomerization to occur in a physiologically relevant setting.^48^ Further exploration of speciofoline and its related diastereomers activity is warranted, due to the high prevalence among commercial chemotypes of kratom and lack of investigation of nonopiate-related activities.^14^

As Angyal et al. discussed briefly, if the time scale in which the interconversion exists is similar to the binding-dissociation events regarding a functionally significant conformational change in the target receptor, then the mechanism of ligand–receptor binding must be reconsidered. While it may be that a specific diastereomer has a higher affinity for an enzyme in terms of drug metabolism, it could very well be that the resulting metabolite undergoes the same interconversions as the spiro-oxindole precursor. The result would be a greater number of metabolites formed, regardless of whether all diastereomer precursors were substrates for the enzyme or if biotransformative modifications fully or partially disrupt stereoisomerization, as is the case with mitragynine pseudoindoxyl, where only three of the four possible forms are observed.^22^ This greatly complicates the chemical landscape of available ligands for receptor binding and the explanation of the overall mechanism(s) of action of kratom.

### Limitations

The clinical translatability of this newly proposed mixture metabolism approach must be noted. Different extraction methods results in varying concentrations and ratios of compounds. While the aim of this model is to consider the interactive effects of multiple substrates on drug-metabolizing enzymes, it does not consider bioavailability and distribution. The substrate mixture for drug-metabolizing enzymes in the body upon consuming kratom would be affected by the bioavailability and distribution of each precursor. Furthermore, the distribution of the metabolite to relevant tissues, such as the blood-brain barrier permeability, cannot be examined in this approach. Lastly, the chosen time points were selected to align with a previously established high-throughput method for drug metabolite identification, with the goal of enabling future application of this model to other medicinal natural product extracts^49,50^ and maximizing observable differences during mixture incubations. Notably, this approach facilitates detection of phenomena such as stereoisomerization of precursors and metabolites, as demonstrated with the incubation of speciofoline. The translatability of relative abundances of metabolites observed in this model toward clinical detection in humans will need to be validated with future experiments.

## Conclusions

The approach used here could be applied to study other natural product mixtures. Based on the workflow described herein, *in silico* prediction of likely metabolite libraries can be generated in an untargeted manner by using LCMS library matching. Analog matches for detected chemicals in natural product extracts by tools like MS2Query enable annotation of similar structural class. These matches likely contain many of the same structural motifs of the true structure, and therefore, adequate mass shifts can be predicted for metabolites using Biotransformer 3.0. Linking precursors to biotransformation metabolites in an untargeted manner would enable the curation of biotransformation libraries for natural products, which can be further validated by experimental detection *in vitro*. Building on previous work in which a similar approach was taken for individual drugs, generation of precursor and metabolite relationships for natural product mixtures would aid in the annotation of compounds in metabolomics data sets.^50^ The translation of the model must be addressed in future studies. Although mitragynine and related alkaloids are known to be bioavailable, not all components of the extract are, potentially convoluting the enzymatic interactive effects observed.

A multilayered molecular networking approach for the generation and detection of plant biotransformation products was developed, enabling the detection and rapid discovery of metabolites directly from a natural product mixture of kratom. Furthermore, the metabolism of speciofoline was reported for the first time. This approach can greatly facilitate understanding of the overall mechanism(s) of action of kratom due to the numerous structurally related precursors and biotransformation products. Use of orthogonal criteria to address the complex chemical landscape of precursors, multiple reaction sights, and the possibility of nonenzymatic-based formation of metabolites can improve understanding of metabolism and activities of multiple kratom chemotypes.

## Experimental Section

### Materials

Pooled human liver S9 fractions and the RapiStart NADPH regeneration system were purchased from Xenotech (Kansas City, MO). Cofactors GSH, PAPS, and acetyl-CoA were purchased from Sigma-Aldrich (Milwaukee, WI), UDPGA from US Biological (Salem, MA), and Alamethicin from Cayman Chemical (Ann Arbor, MI). Mitragynine, isotopically labeled mitragynine-d_3_, corynoxeine, and paynantheine (>99%) were purchased from Cayman Chemical. 7-Hydroxymitragynine certified reference material was purchased from Sigma-Aldrich. Purified speciofoline was provided as previously described.^10^ Extracts K52 and K49 were authenticated by DNA barcoding of the plastid intergenic spacer *trnH-psbA*, *matK*, and the nuclear ribosomal internal transcribed spacer (ITS) regions as previously described.^10^ Extract KTP (lyophilized kratom tea extract) was chemically authenticated through comparison to the K49 and K52 extracts.

### Liver S9 Incubation

Incubations were done according to previous methods, with modifications to incubation concentrations.^49,50^ S9 fractions (20 mg/mL protein) were aliquoted into 0.5 mL microcentrifuge tubes, stored at −80 °C, and then thawed at room temperature prior to use. Standards were prepared in DMSO and further diluted in water prior to addition in the well for a final testing concentration of 10 μM. Extracts were prepared in water at a final concentration in the well at 500 μg/mL, which was determined by the method of standard addition (data not shown) to be an approximately equal concentration in extract K52 as 10 μM mitragynine used in single-compound incubations (0.85%w:w mitragynine). All substrate materials were centrifuged briefly (10,000*g*, 5 min) after dilution and prior to addition to test wells to ensure no particulates were present. Isotopically labeled mitragynine was spiked in K52 kratom extract at a final concentration of 1 μM. Individual compounds were incubated at 10 μM. The NADPH regenerating system was reconstituted with 3.5 mL of water to make a final volume of 5 mL. The 4× cofactor stock included 10 mM UDPGA, 2 mM GSH, 2 mg/mL PAPS, 0.1 mM acetyl-CoA, and NADPH regenerating system (1 mM NADP, 5 mM glucose-6-phosphate, 1 unit glucose-6-phosphate dehydrogenase). The S9 fraction was diluted 10-fold in water prior to the addition of buffer (0.2 M Tris-Cl, pH 7.5, 2 mM MgCl_2_) and test article in equal amounts, then preincubated at 30 °C for 5 min. To start the reaction, 15 μL of 4× cofactor stock was added, and the 96-well plate was incubated at 30 °C for 0, 2, 6, and 24 h. The final S9 protein concentration was 500 μg/mL. To terminate the reaction, a 3-fold volume of ice-cold acetonitrile was added. The plate was covered with parafilm, vortexed, and frozen at −20 °C to precipitate insoluble materials, such as proteins. After thawing and centrifugation of the incubation plate, the supernatants were transferred into polypropylene autosampler vials and stored at −20 °C until instrumental analysis.

### LCMS Analysis

The analytical platform consisted of a Thermo Scientific Vanquish UHPLC instrument coupled to an Orbitrap ID-X mass spectrometer with an ESI source operated in positive mode. Spray voltage was set to 3.5 kV, with a vaporizer temperature of 275 °C for normal phase conditions and 350 °C for reverse phase. Profile MS^1^ scans collected at 60,000 resolution and centroided MS^2^ scans collected at 15,000 resolution. For the reverse phase, mobile phases of H_2_O (A) and MeOH (B), both acidified with 0.1% formic acid, were used with a Hypersil GOLD column (50 mm × 2.1 mm, 1.9 μm particle size; Thermo Scientific, Waltham, MA) with an Acquity HSS T3 guard (5.0 mm × 2.1 mm, 1.8 μm particle size; Waters corporation Milford, MA) and 0.35 mL/min flow rate. Chromatographic conditions start with a hold at 15% B for 0.75 min, increasing to 60% B over 6 min, increasing to 99% B over 0.75 min, followed by a hold at 99% B for 2.5 min before returning to starting conditions. For hydrophilic interaction liquid chromatography (HILIC), mobile phases consisted of H_2_O (A) and ACN (B), both acidified with 0.1% formic acid, with a Waters Acquity BEH Amide column (2.1 mm × 100 mm, 1.7 μm particle size) flowing at 0.20 mL/min. Chromatographic conditions started at 90% B and were held for 1.5 min, decreasing to 20% B over 6 min, followed by a hold at 20% B for 2.5 min.

### LCMS Data Processing and Extraction

Raw data were converted to mzML format using Proteowizard MSconvert^51^ prior to extraction in Mzmine version 4.5.37.^52^ Batch files with all extraction parameters can be found in the Supporting Files. Briefly, mass detection was performed for MS^1^ and MS^2^ scans followed by chromatogram building, smoothing, local minimum feature resolving, and ^13^C isotope filtering. Features were aligned and filtered prior to spectral molecular networking using both modified cosine score (threshold of 0.65) and MS2Deepscore with a minimum similarity of 85%.^53^ Feature tables, MS^2^ spectra, and molecular network files were exported and further analyzed using Python scripts. Principal component analysis (PCA) of extracts was performed in Mzmine based on pareto scaled peak areas.

### *In Silico* Metabolite Predictions

Biotransformer 3.0 running batch files generated in Python from precursor SMILES was used for *in silico* predictions.^35^ A manually generated input table was used for the individual compounds. Two rounds of “all human” biotransformation were selected. A list of possible adduct *m*/*z* values for metabolite monoisotopic masses was calculated and used to query the feature table.

### Data Analysis

Anaconda version 24.7.1 with Python version 3.10.14 and Pandas packages were used for feature table data analysis. Log_2_ transformed peak area features tables were analyzed by ANOVA with FDR-corrected *p*-values (*p* < 0.05) using Statsmodels and SciPy packages to assess the effect of time on the variation of each features’ peak area. Feature subtraction was performed to remove significant features found in vehicle control. Additional statistics and graphing were performed in GraphPad Prism version 10.4.2.

### Networking

The NetworkX package was used for the construction of graphs in combination with Cytoscape version 3.10.2. *In silico* networks were built from the Biotransformer 3.0 outputs for each compound, and the Cytoscape plugin (Chemoinformatics tools) was used for graphing predicted structures. Nodes in the networks were annotated matching the following criteria: Predicted Mass (Biotransformer), significant FDR-corrected *p*-value (*p* < 0.05), piecewise slope between time points, and mass defect filtering. These were combined with second-degree neighbor precursor MS^2^ networks output from Mzmine, to incorporate MS^2^ spectral similarity as measured by both modified cosine and MS2Deepscore.^53^

## Supplementary Material





## Notes

Metabolite annotations (Tables S1 and S2 and Figures S1–S7 and S10) and MS^2^ spectra (Figures S8, S9, and S11–S27) (PDF)
Mzmine parameter batch files (ZIP)
