# Supplementary material for: Mixtures Biotransformation: Multilayer Molecular Networking of Kratom Liver Metabolites
Source: J Nat Prod. 2026 Feb 10;89(2):433–43. doi: 10.1021/acs.jnatprod.5c01235 (PMC12954754; doi:10.1021/acs.jnatprod.5c01235)
Supplement: Supplementary file 1 [file np5c01235_si_001.pdf]

Supporting Information

Mixtures Biotransformation: Multi-Layer Molecular Networking of Kratom Liver Metabolites

Authors
William J. Crandall[a], Jaclyn Weinberg[a], Ken Liu[b], Choon-Myung Lee[c], Grant Singer[a], Edward T. Morgan[d], Dean P. Jones[c]*, Cassandra L. Quave[e,f,g]*.

[a] Molecular and Systems Pharmacology, Emory University, 201 Dowman Drive, Atlanta, GA, 30322, United States.
[b] Department of Chemistry, Emory University, 201 Dowman Drive, Atlanta, GA, 30322, United States.
[c] Department of Medicine: Pulmonary, Allergy, Critical Care, and Sleep Medicine, Emory University, 201 Dowman Drive, Atlanta, GA, 30322, United States.
[d] Department of Pharmacology & Chemical Biology Professor Emeritus, Emory University, 201 Dowman Drive, Atlanta, GA, 30322, United States.
[e] Department of Dermatology, Emory University, 201 Dowman Drive, Atlanta, GA, 30322, United States.
[f] Center for the Study of Human Health, Emory University, 201 Dowman Drive, Atlanta, GA, 30322, United States.
[g] Emory University Herbarium, Emory University, 1462 Clifton Rd NE, Room 102, Atlanta, GA, 30322, United States.

*Email: CLQ: cquave@emory.edu; DPJ: dpjones@emory.edu

Table of Contents

Table S1: Metabolites of Mitragynine, Paynantheine, and Corynoxeine detected by HILIC chromatography. 3

Table S2: Metabolites of Mitragynine and Speciofoline detected by C18 chromatography. 4

Figure S1: Speciofoline network comparison. 5

Figure S2: MSn fragmentation of labeled and unlabeled phase II mitragynine metabolite.......... 6

Figure S4: Extract chemotype. 8

Figure S5: Combined mitragynine and speciofoline detected metabolites........ 9

Figure S7: Time course of phase II m/z 591 metabolite of mitragynine........ 10

Figure S8: MS2 spectrum of 9-O-demethylmitragynine (9-hydroxycorynantheidine). 11

Figure S9: MS2 spectrum of 16-carboxymitragynine........ 12

Figure S10: Chromatographic separation of Mitragynine and its diastereomers........ 12

Figure S11: Theoretical convergent mechanism of mitragynine diastereomer metabolites. 13
Figure S12: Comparison of speciofoline isomers in extract to stereoisomerization controls. 14
Figure S13: Proposed metabolic pathway for speciofoline.. 15
Figure S14: MS2 spectra of mitragynine linked to Table S2.. 16
Figure S15: MS2 spectra of 7OH-mitragynine linked to Table S2. 17
Figure S16: MS2 spectra of mitragynine metabolite linked to Table S2. 18
Figure S17: MS2 spectra of mitragynine metabolite linked to Table S2. 19
Figure S18: MS2 spectra of mitragynine metabolite linked to Table S2. 20
Figure S19: MS2 spectra of speciofoline linked to Table S2.. 21
Figure S20: MS2 spectra of speciofoline metabolite linked to Table S2. 22
Figure S21: MS2 spectra of speciofoline metabolite linked to Table S2. 23
Figure S22: MS2 spectra of speciofoline metabolite linked to Table S2. 24
Figure S23: MS2 spectra of speciofoline metabolite linked to Table S2. 25
Figure S24: MS2 spectra of speciofoline metabolite linked to Table S2. 26
Figure S25: MS2 spectra of speciofoline metabolite linked to Table S2. 27
Figure S26: MS2 spectra of speciofoline metabolite linked to Table S2. 28
Figure S27: MS2 spectra of mitragynine metabolite linked to Table S2. 29

Table S1: Metabolites of Mitragynine, Paynantheine, and Corynoxeine detected by HILIC chromatography.

Metabolite ID	m/z	rt (min)	Δ Mass (Da)	Δ Formula	Detected in Mixture*
**Mitragynine (MG)**	399.2280	1.58	-	-	Yes
Dehydro-MG	397.2127	1.51	-2.0157	- H2	
OH-MG	415.2231	1.53	+15.9949	+O	Yes
9-O-demethyl-MG/ 16-carboxy-MG	385.2124	1.53	-14.1057	-CH2	Yes
M1	369.2176	1.53	-30.0106	-CH2O	-
M2	401.2079	1.99	+1.9792	-CH2, +O	-
OH-MG-G	591.2554	3.91	+192.0270	+C6H8O7	Yes
M3	563.2605	4.36	+164.0321	+C5H8O6	-
**Paynantheine (PAY)**	397.2125	1.54	-	-	Yes
9-O-demethyl-PAY/ 16-carboxy-PAY	383.1968	1.51	-14.1057	-CH2	Yes
Dehydro-PAY	395.197	1.53	-2.0157	- H2	-
OH-PAY	413.2075	1.54	+15.9949	+O	-
M4	399.1920	1.58	+1.9792	-CH2, +O	-
M5	431.2185	2.12	+34.0054	+H2O2	-
OH-PAY-G	589.2399	3.93	+192.027	+C6H8O7	-
M6	561.2447	4.25	+164.0321	+C5H8O6	-
**Corynoxeine (COR)**	383.1971	1.54	-	-	Yes
Dehydro-COR	381.1817	1.24	-2.0157	- H2	-
Dehydro-COR	381.1816	1.54	-2.0157	- H2	-
16-carboxy-COR	369.1818	1.56	-14.1057	-CH2	-
OH-COR	399.1921	1.65	+15.9949	+O	-
M7	417.2027	2.40	+34.0054	+H2O2	-
M8	417.2028	2.54	+34.0054	+H2O2	Yes
M9	433.1978	2.98	+50.0003	+H2O3	-
M10	433.1979	3.25	+50.0003	+H2O3	-
OH-COR-G	575.2252	4.08	+192.0270	+C6H8O7	-

* Detection of metabolite after mixtures metabolism KTP extract.

-G = glucuronide

Table S2: Metabolites of Mitragynine and Speciofoline detected by C18 chromatography.

Metabolite ID	m/z	rt (min)	Δ Mass (Da)	Δ Formula	Detected in Mixture*	MS2‡
**Mitragynine** (MG)	399.2262	5.23	-	-	Yes	S14
7OH-MG-G	591.2525	3.40	+192.0263	+C6H8O7	-	-
M11^	433.2318	3.77	+34.0056	+H2O2	-	S27
7OH-MG	415.2213	3.77	+15.9951	+O	Yes	S15
M12	447.2474	3.78	+48.0212	+CH4O2	-	-
M13	415.2213	3.89	+15.9951	+O	Yes	S16
9-O-demethyl-MG	385.2109	4.01	-14.0153	-CH2	Yes	S8
OH-MG-G	591.2525	4.04	+192.0263	+C6H8O7	-	-
M14	433.2319	4.10	+34.0057	+H2O2	-	-
M15	387.2265	4.12	-11.9997	-C	-	-
M16	415.2214	4.24	+15.9952	+O	Yes	S17
16-carboxy-MG	385.2109	4.73	-14.0153	-CH2	-	S9
M17	415.2214	5.02	+15.9952	+O	Yes	-
M18	415.2213	5.30	+15.9951	+O	-	S18
M19	415.2214	5.37	+15.9952	+O	-	-
M20	415.2212	6.40	+15.9950	+O	Yes	-
Speciofoline (SP)	401.2054	4.76	-	-	Yes	S19
M21	387.1900	2.81	-14.0154	-CH2	-	-
M22	387.1900	2.99	-14.0154	-CH2	-	S20
M23	387.1899	3.15	-14.0155	-CH2	-	S21
M24	387.1899	3.30	-14.0155	-CH2	-	S22
M25	415.1848	3.43	+13.9794	- H2, +O	Yes	-
M26	417.2004	3.73	+15.9950	+O	Yes	-
16-carboxy-SP	387.1899	3.93	-14.0155	-CH2	Yes	S23
M27	417.2004	4.01	+15.9950	+O	-	S24
M28	399.1899	4.07	-2.0155	- H2	Yes	-
M29	399.1899	4.17	-2.0155	- H2	-	S25
M30	417.2003	4.22	+15.9951	+O	-	S26
M31	415.1848	4.30	+13.9794	- H2, +O	-	-

*Detection of metabolite after mixtures metabolism of K52, K49 or KTP extracts.

‡ Supplemental figure code Figure () for MS2 if collected by data dependent acquisition.

^ M11 is likely an adduct of 7-hydroxymitragnyine.

-G = glucuronide

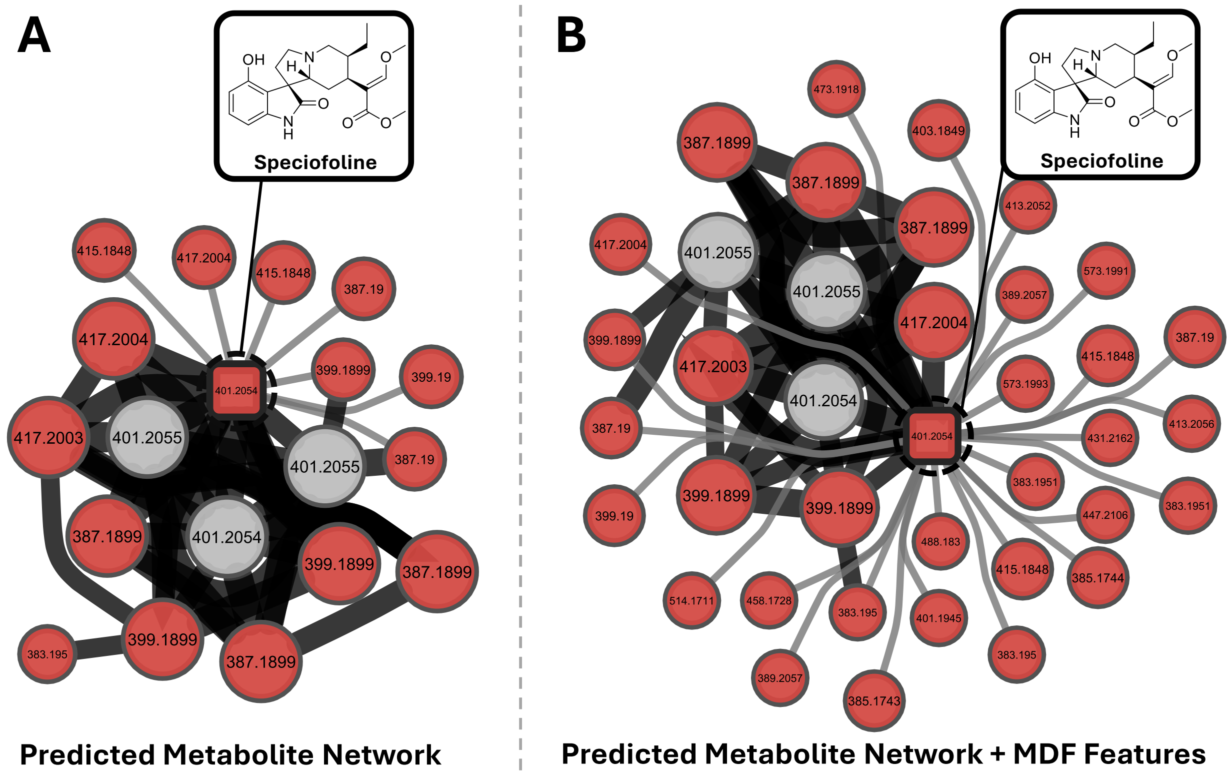

Figure S1: Speciofoline network comparison. A) Annotated features networked without inclusion of mass defect filtering (MDF) when incubating with speciofoline. B) A larger number of annotated features networked with inclusion of MDF is observed.

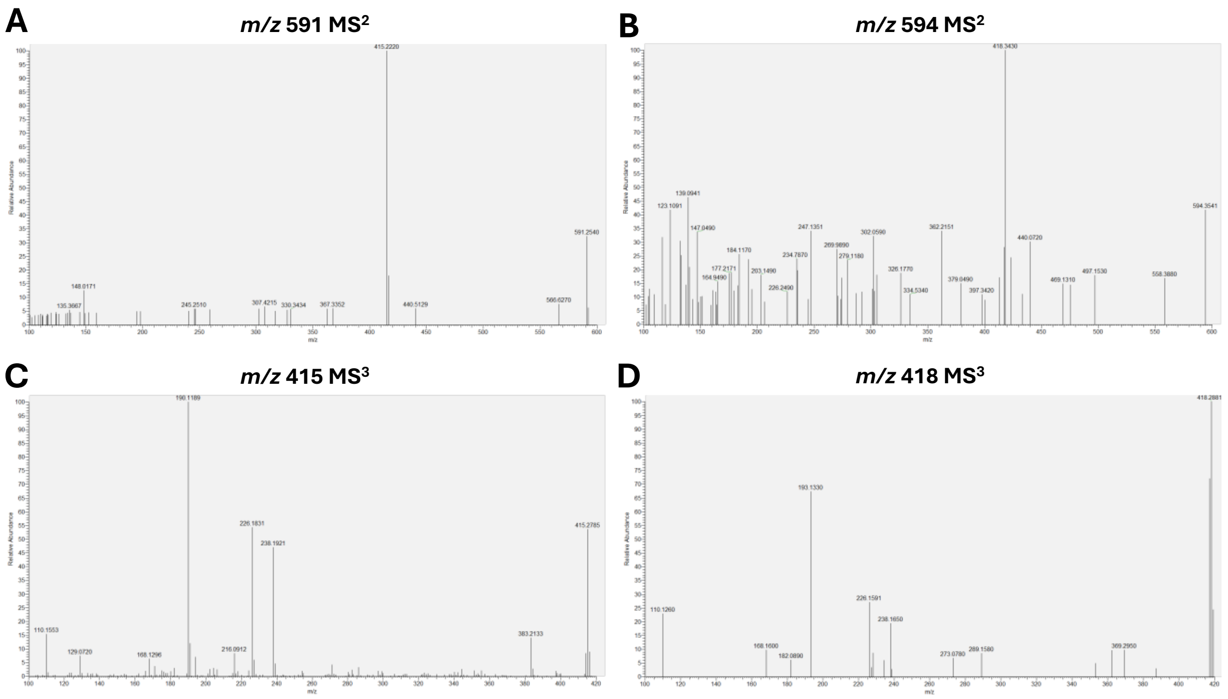

Figure S2: MSn fragmentation of labeled and unlabeled phase II mitragynine metabolite. A) MS2 fragmentation of m/z 591 metabolite of mitragynine at 15,000 resolution detected in the orbitrap with m/z 415.2220 main product ion corresponding to mono-oxidated metabolite of mitragynine. **B)** MS2 fragmentation of D3 – isotopically labeled m/z 594 metabolite detected in the ion trap with corresponding labeled mono – oxidated metabolite of mitragynine at m/z 418.3430. **C)** MS3 fragmentation of m/z 415 ion showing characteristic m/z 190, 226, and 238 product ions detected in the ion trap. **D)** MS3 fragmentation of labeled m/z 418 ion showing the same m/z 226 and 238 product ions, and m/z 193 product ion corresponding to the fragment containing D3-labeled methoxy on the indole ring detected in the ion trap.

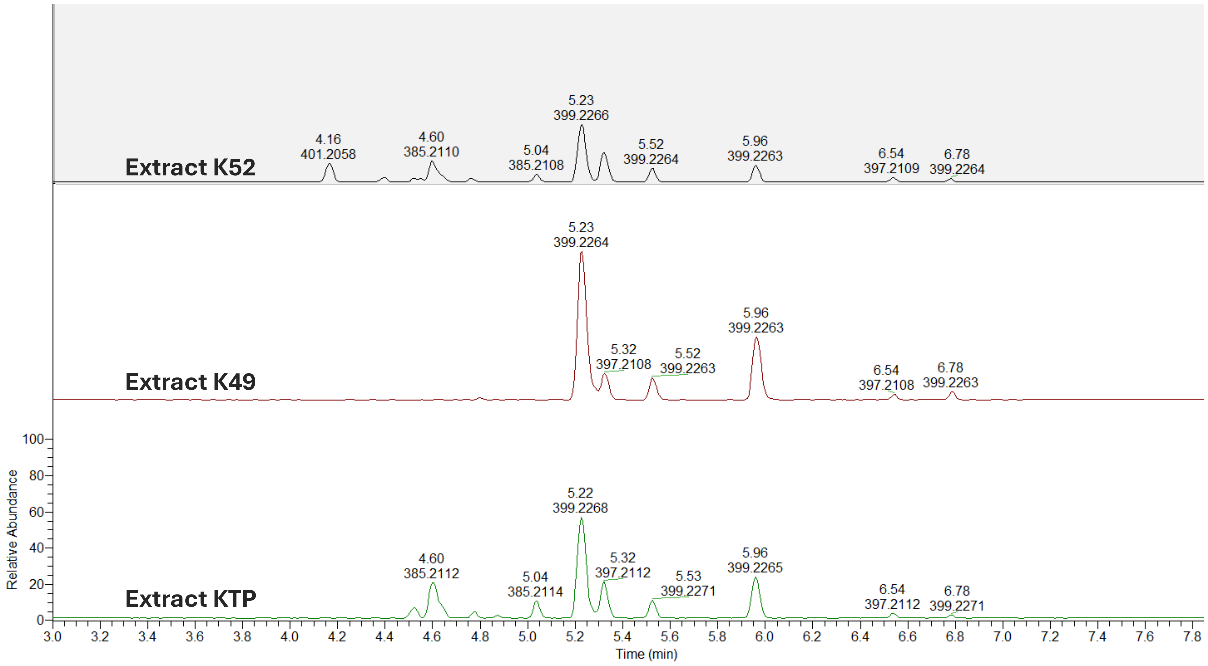

Figure S3: Base Peak chromatograms of each Kratom extract fixed to the same scale.

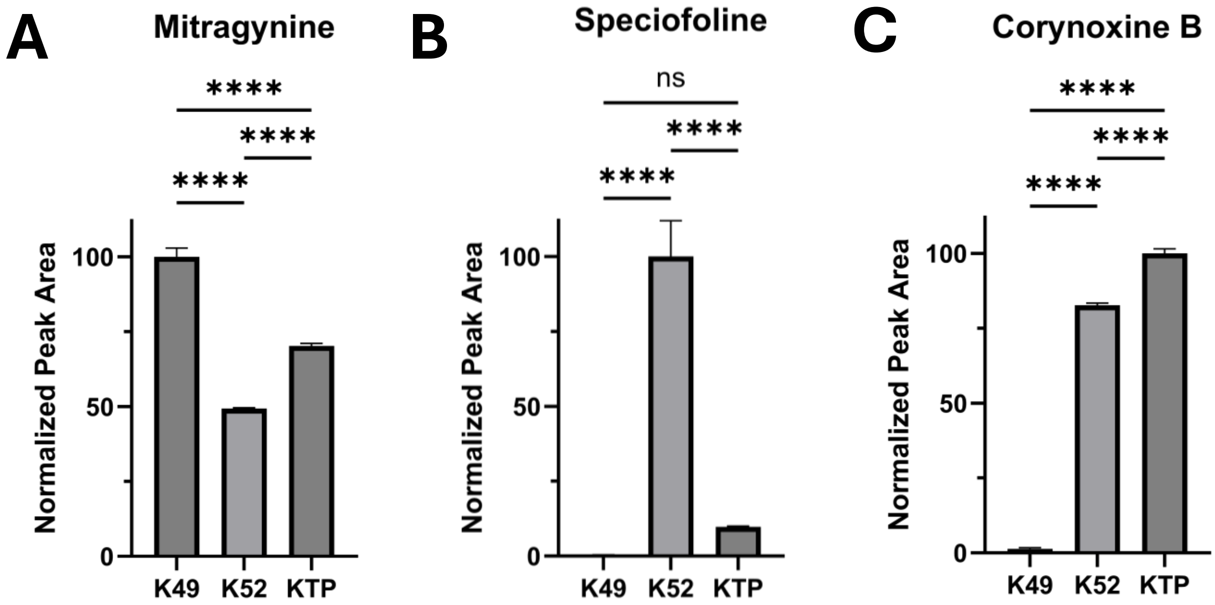

D

	Extract		
**Compound ID**	**K49**	**K52**	**KTP**
Mitragynine	100%	49.3%	70.2%
Speciofoline	0.362%	100%	9.77%
Corynoxine B*	1.31%	82.7%	100%

Figure S4: Extract chemotype. A comparison of the relative abundances of mitragynine **(A)**, speciofoline **(B)** and corynoxine B* **(C)** in each extract normalized to the most abundant peak area. Comparisons for each m/z between extract type conducted by one way Anova with Tukey's post hoc (ns = not significant, **** p <0.0001). **D)** A summary of mean relative abundances reported in panels A-C. *Corynoxine B was annotated through MS2 library matching.

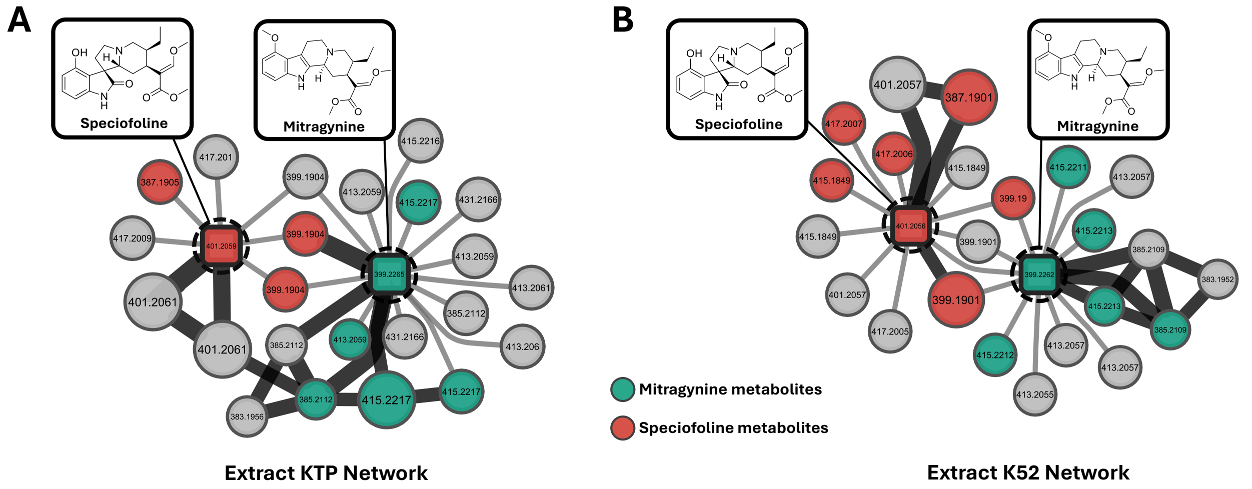

Figure S5: Combined mitragynine and speciofoline detected metabolites in mixtures of KTP(A) and K52 (B). Colored nodes are those that were confirmed by m/z and retention time to metabolites formed for incubating individual standards for each compound respectively.

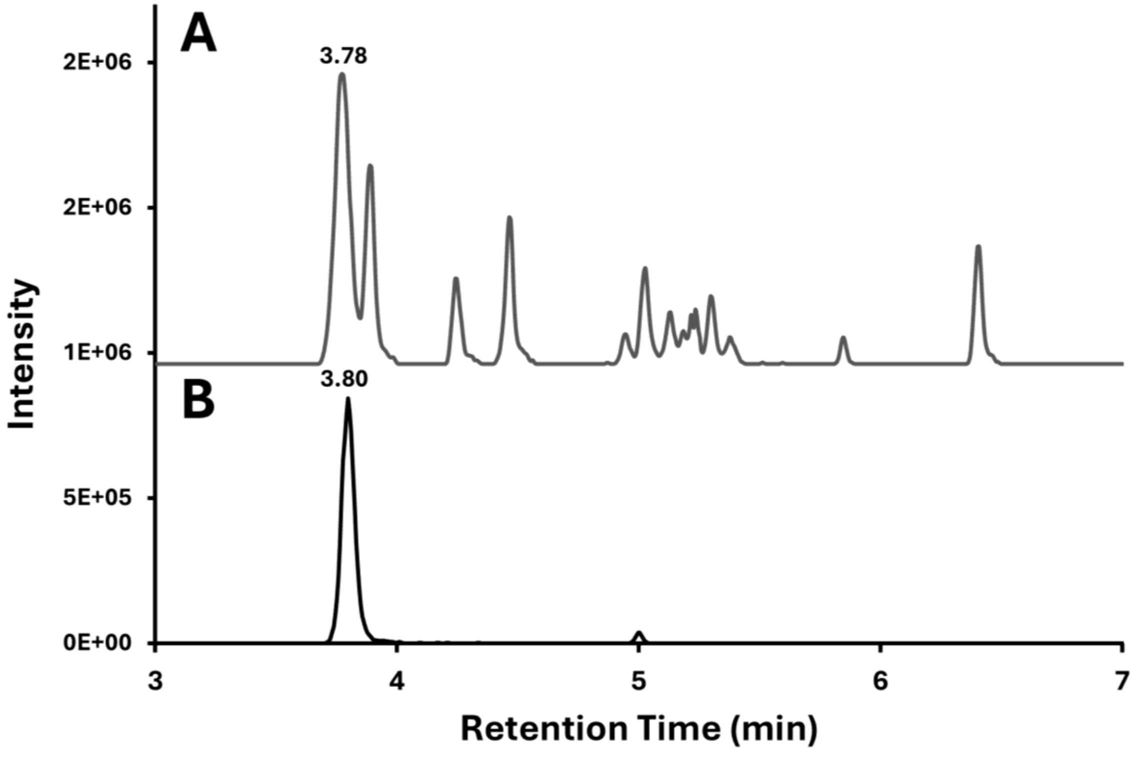

Figure S6: 7-hydroxymitragynine standard comparison. A) Mono-oxidative metabolites detected upon incubation of mitragynine. **B)** Matching retention time to a standard of 7-hydroxymitragynine.

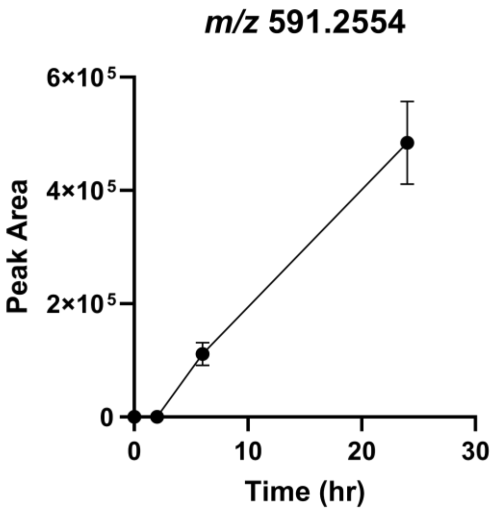

Figure S7: Time course of phase II m/z 591 metabolite of mitragynine. Peak areas of mitragynine metabolite m/z 591.2554 hypothesized to be OH-mitragynine-glucuronide. Time points 0 and 2 hours were not detected. The phase II metabolite increases in abundance over the 6 to 24 hr time points.

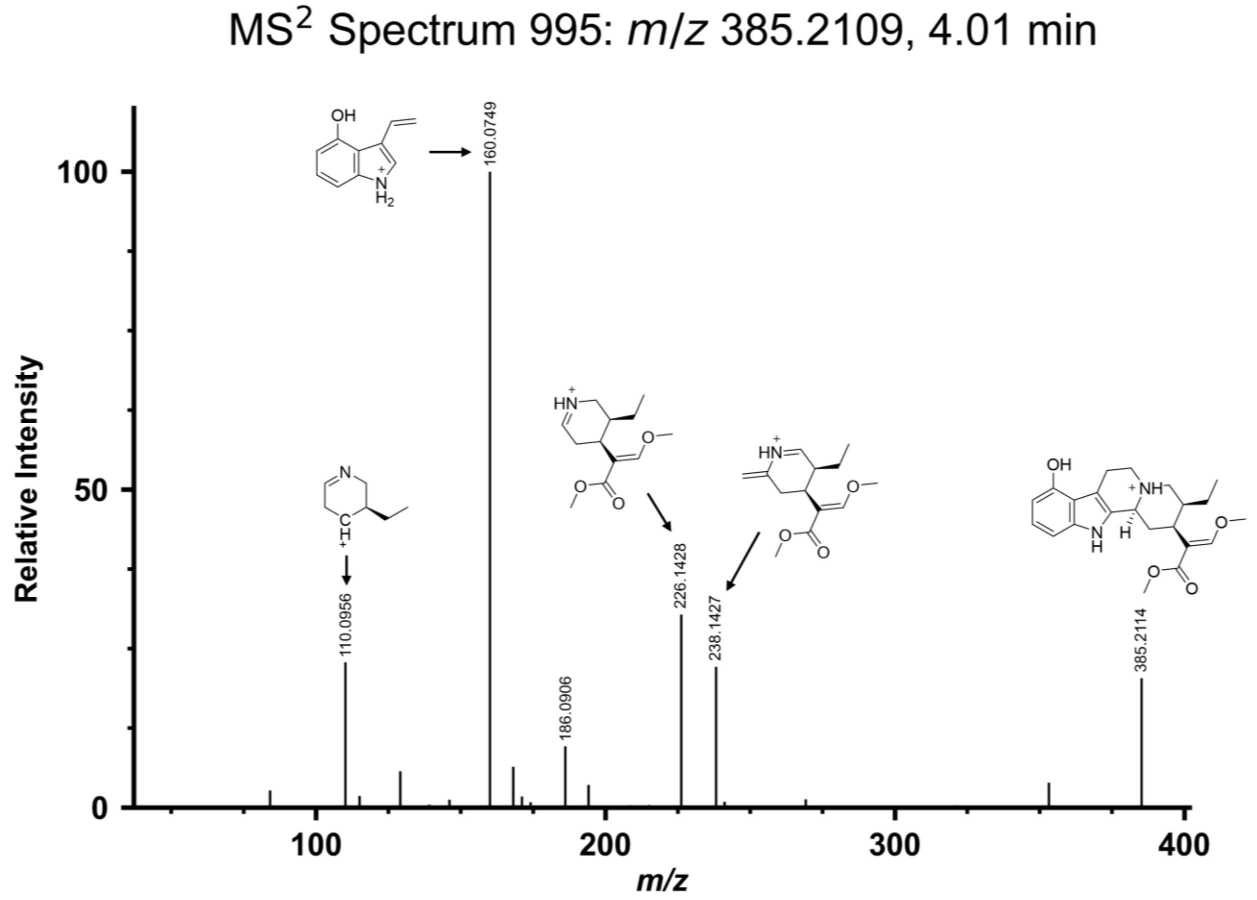

Figure S8: MS2 spectrum of 9-O-demethylmitragynine (9-hydroxycorynantheidine).

Structures for precursor metabolite adduct was annotated along with possible fragment structures.

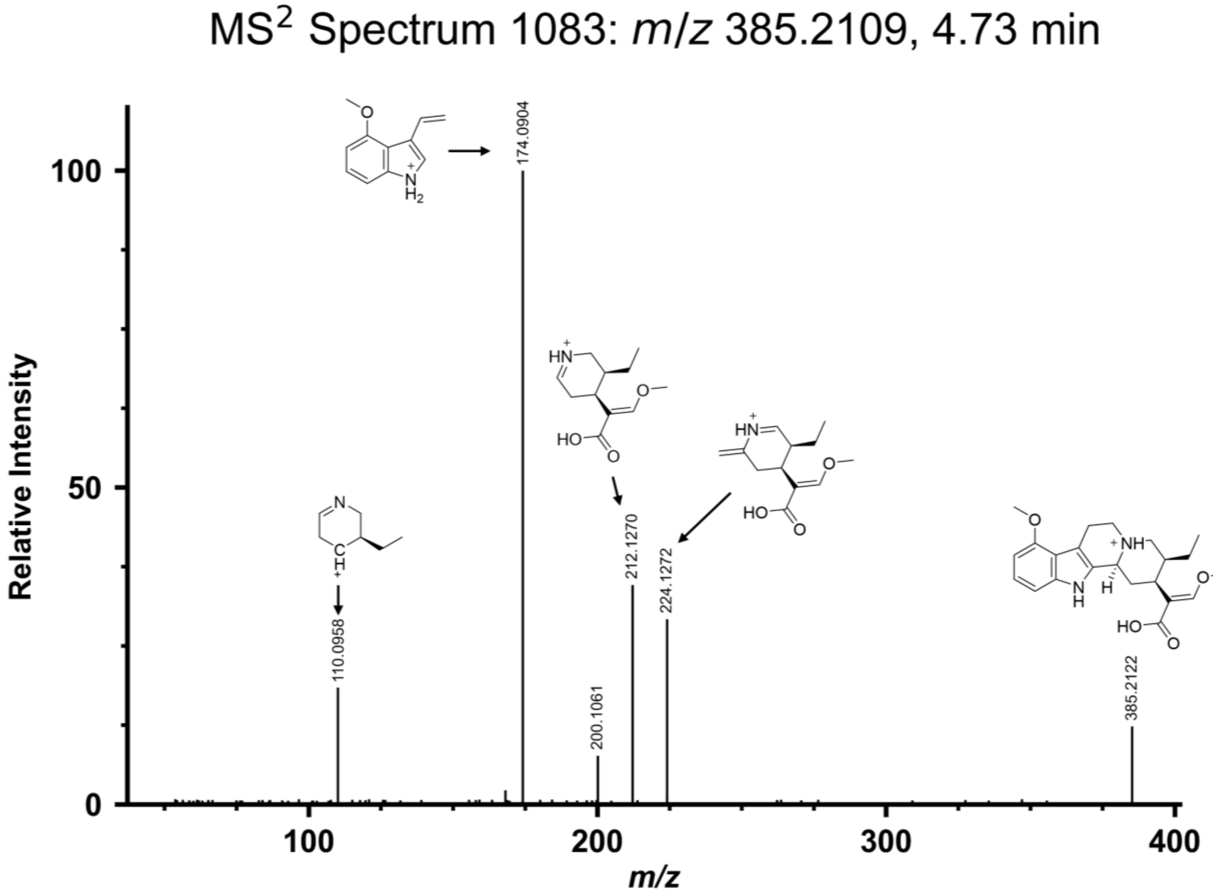

Figure S9: MS2 spectrum of 16-carboxymitragynine.

Structures for precursor metabolite adduct was annotated along with possible fragment structures.

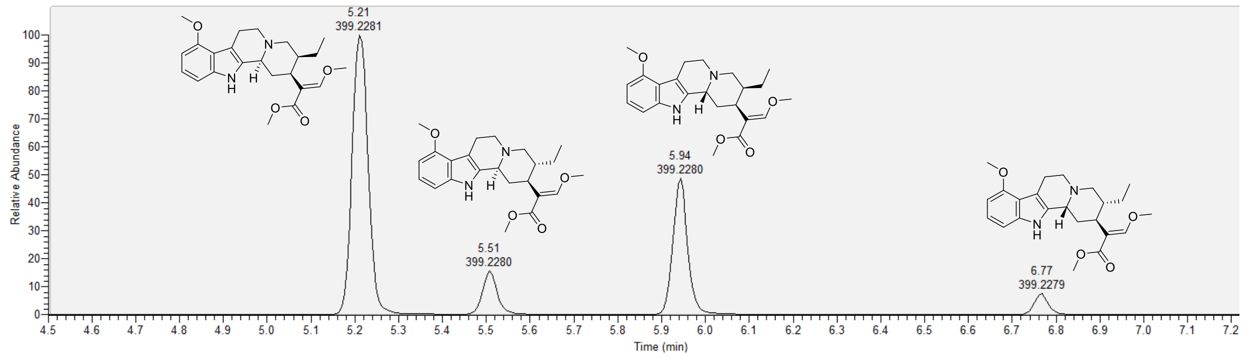

Figure S10: Chromatographic separation of Mitragynine and its diastereomers.

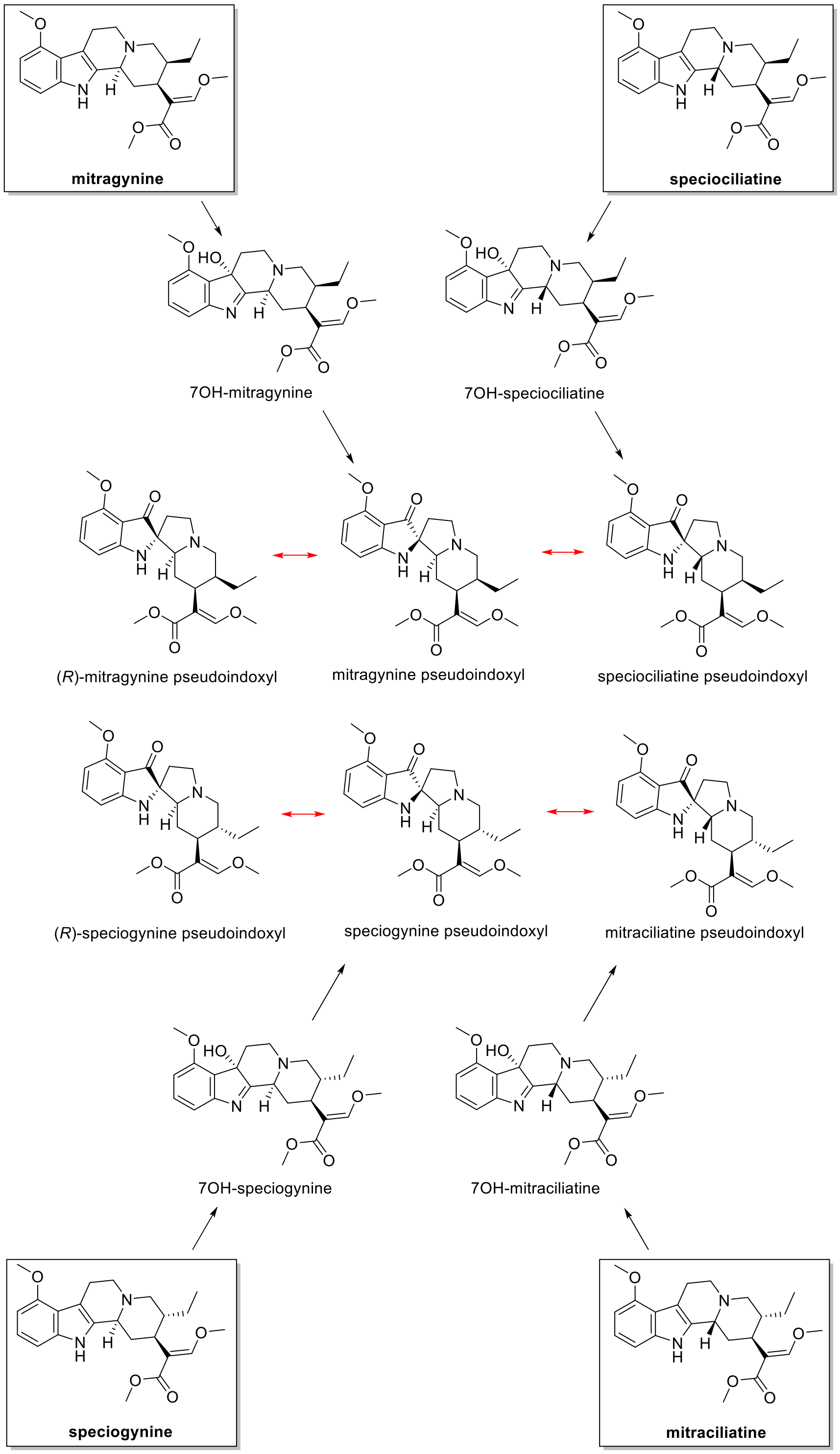

Figure S11: Theoretical convergent mechanism of mitragynine diastereomer metabolites. Red arrows indicate non-enzymatic stereoisomerization. Boxed structures are mitragynine and related diastereomers found in kratom. Spiro carbon of mitragynine pseudoindoxyl and speciogynine pseudoindoxyl are named based on their spiro carbon configuration (R).

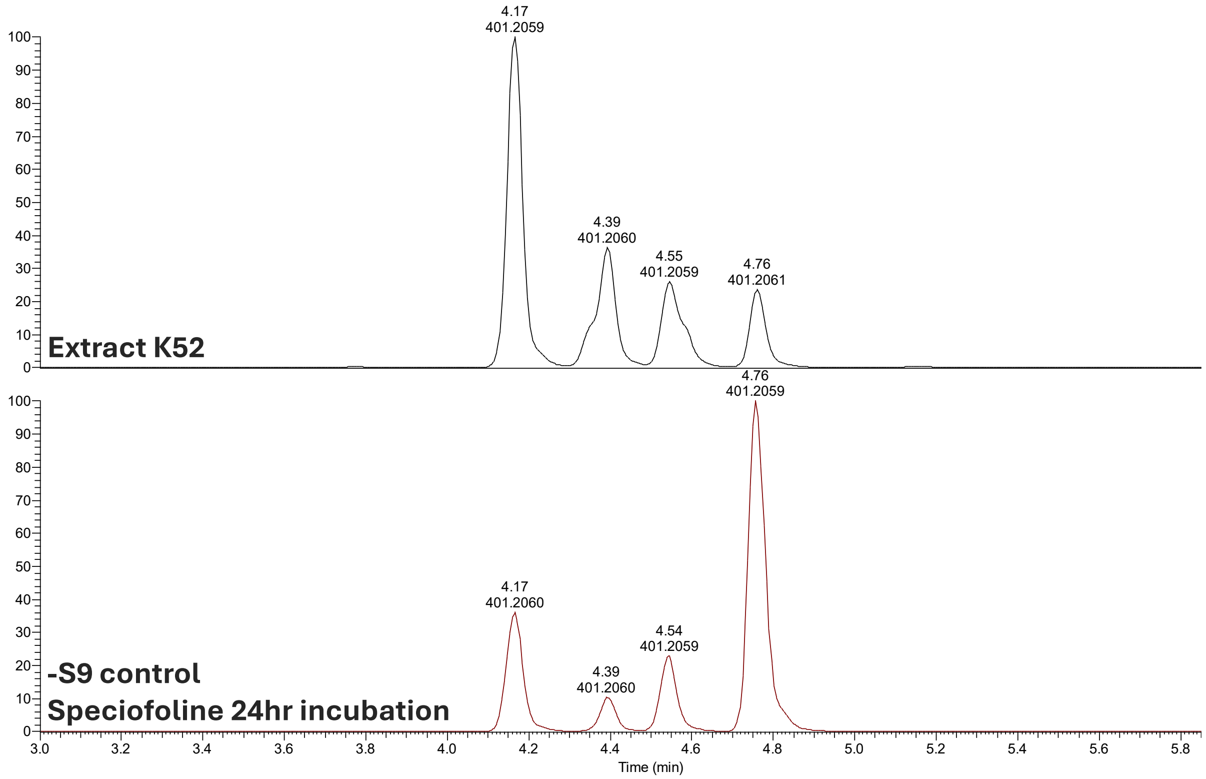

Figure S12: Comparison of speciofoline isomers in extract to stereoisomerization controls. Kratom extract K52 (top) extracted ion chromatogram shows the same diastereomers of speciofoline (4.76 min) as those observed when incubating speciofoline with heat inactivated S9 fractions for 24 hr (bottom).

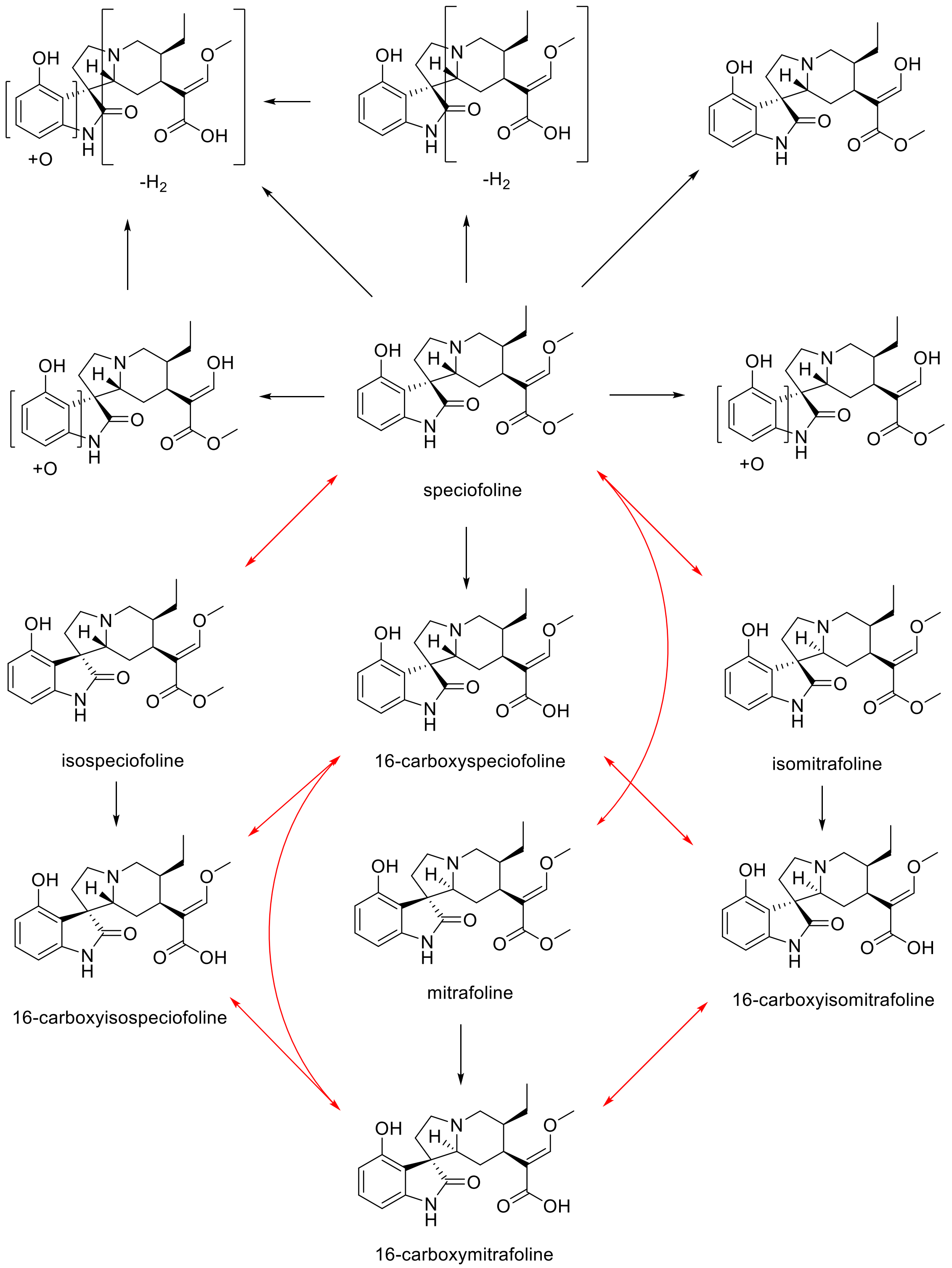

Figure S13: Proposed metabolic pathway for speciofoline. Proposed metabolite structures for speciofoline based on data collected in this study. Red arrows indicate the possibility of stereoisomerization which can occur non-enzymatically.

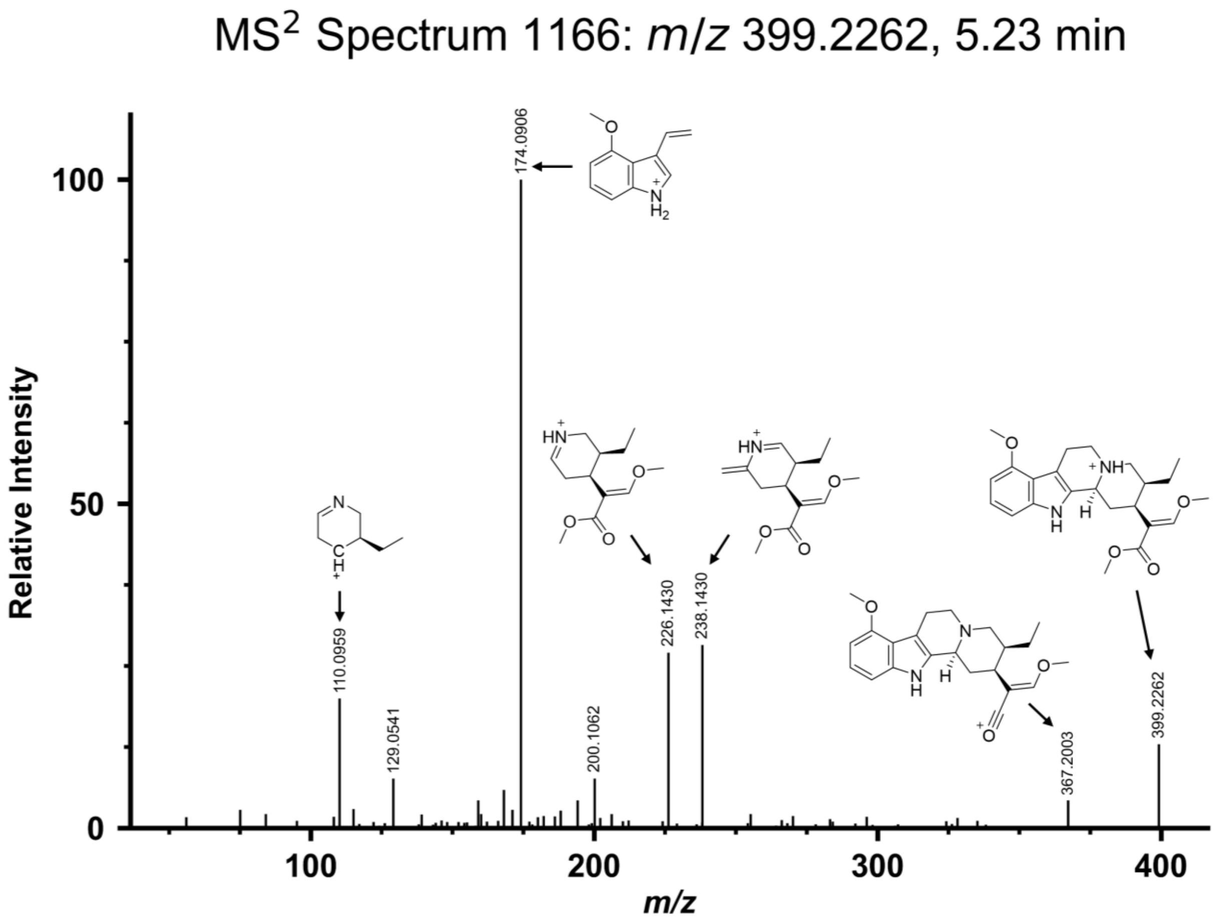

Figure S14: MS2 spectra of mitragynine linked to Table S2. The structure for the precursor adduct was annotated along with possible fragment structures.

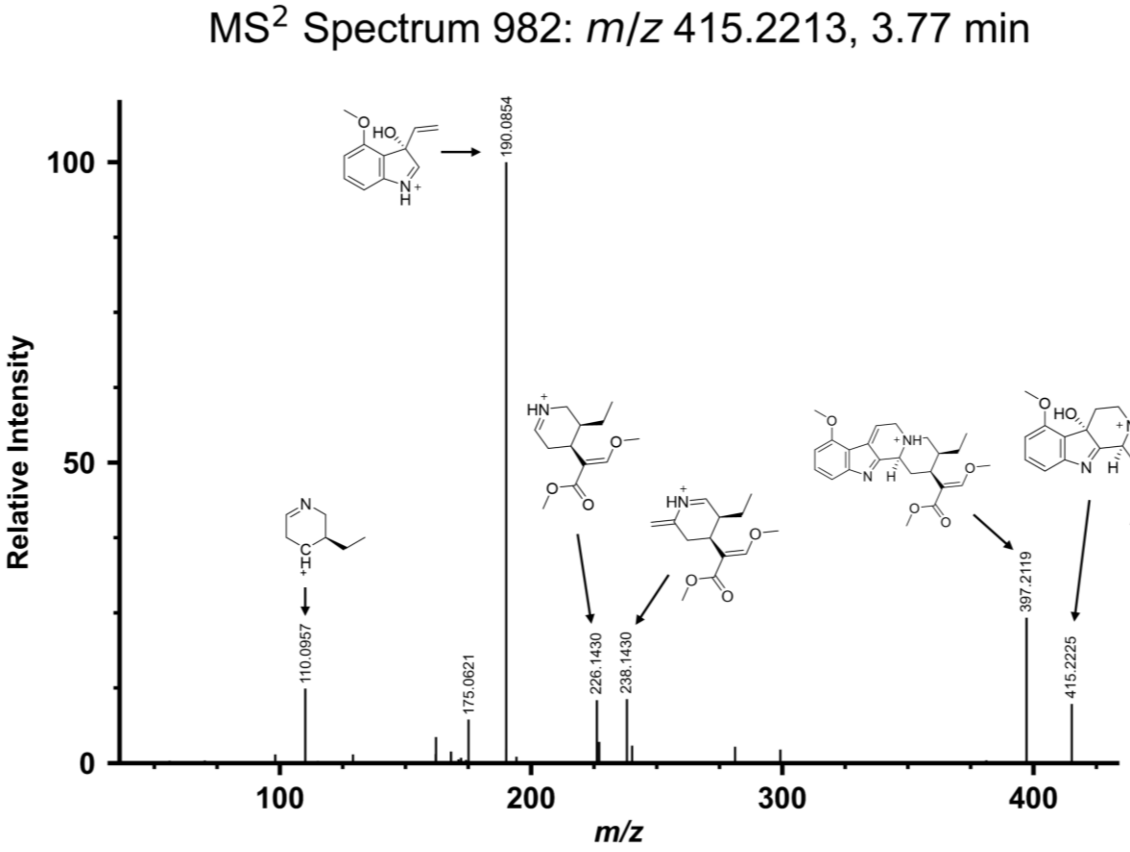

Figure S15: MS2 spectra of 7OH-mitragynine linked to Table S2. The structure for the precursor metabolite adduct was annotated along with possible fragment structures. The m/z 397 product ion is a particular marker for C7 hydroxylation rather than hydroxylation on the benzene ring of the indole.

MS^2 Spectrum 990: *m/z* 415.2213, 3.89 min

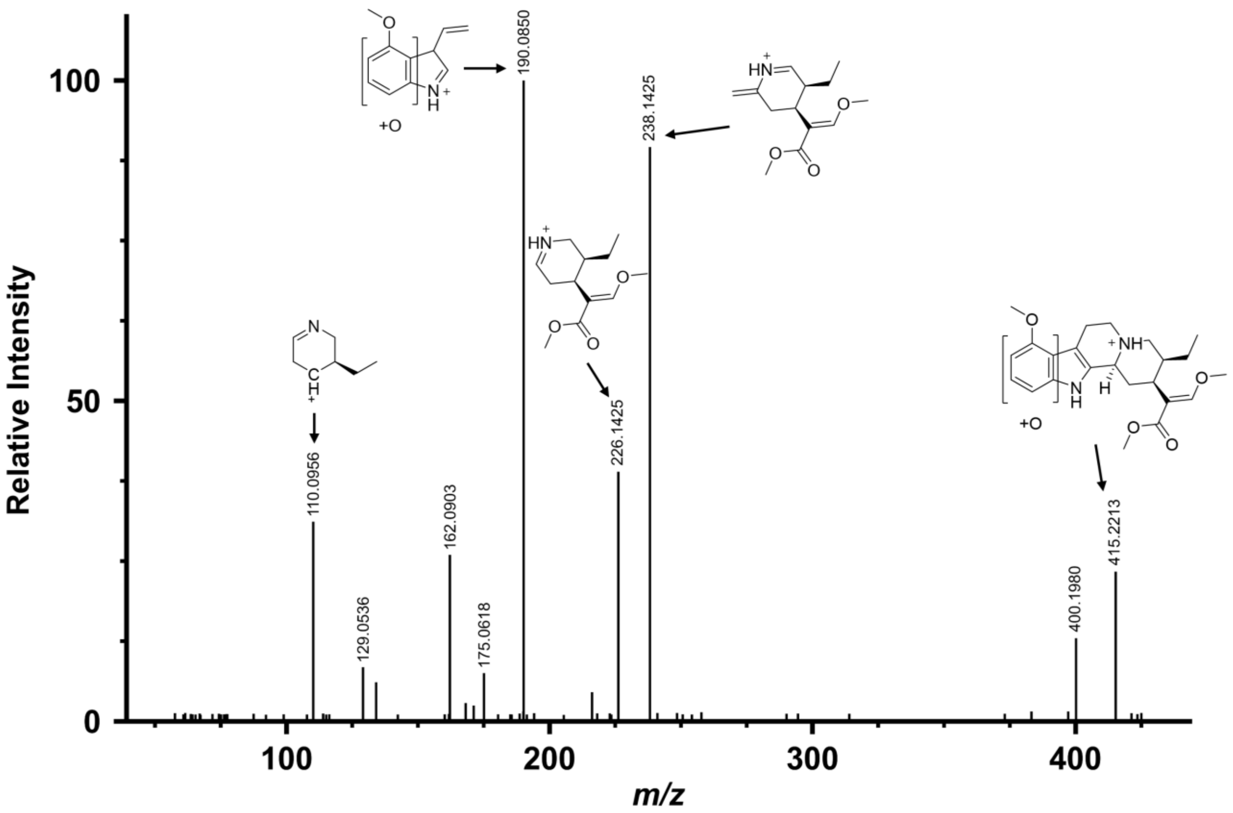

Figure S16: MS2 spectra of mitragynine metabolite linked to Table S2. The structure for the precursor metabolite adduct was annotated along with possible fragment structures.

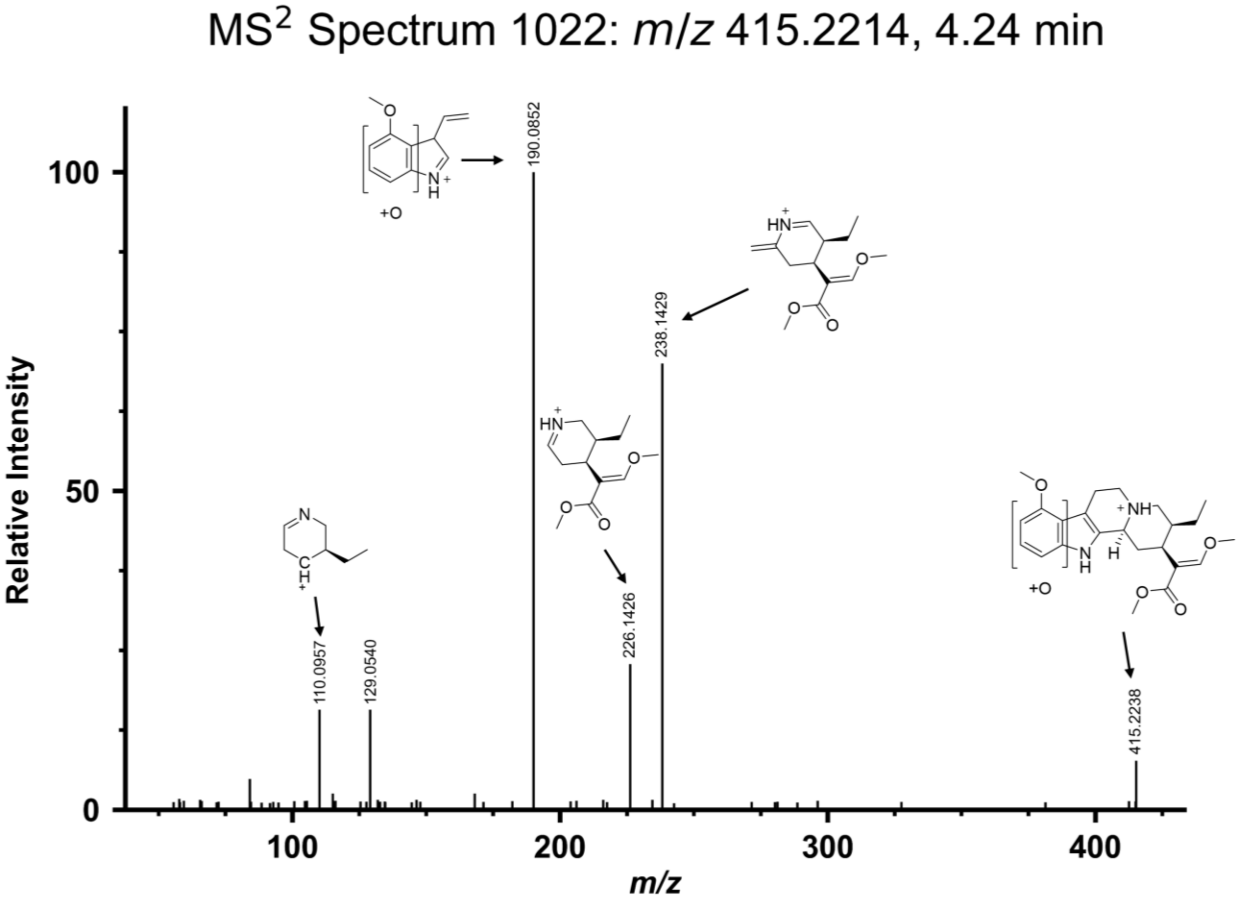

Figure S17: MS2 spectra of mitragynine metabolite linked to Table S2. The structure for the precursor metabolite adduct was annotated along with possible fragment structures.

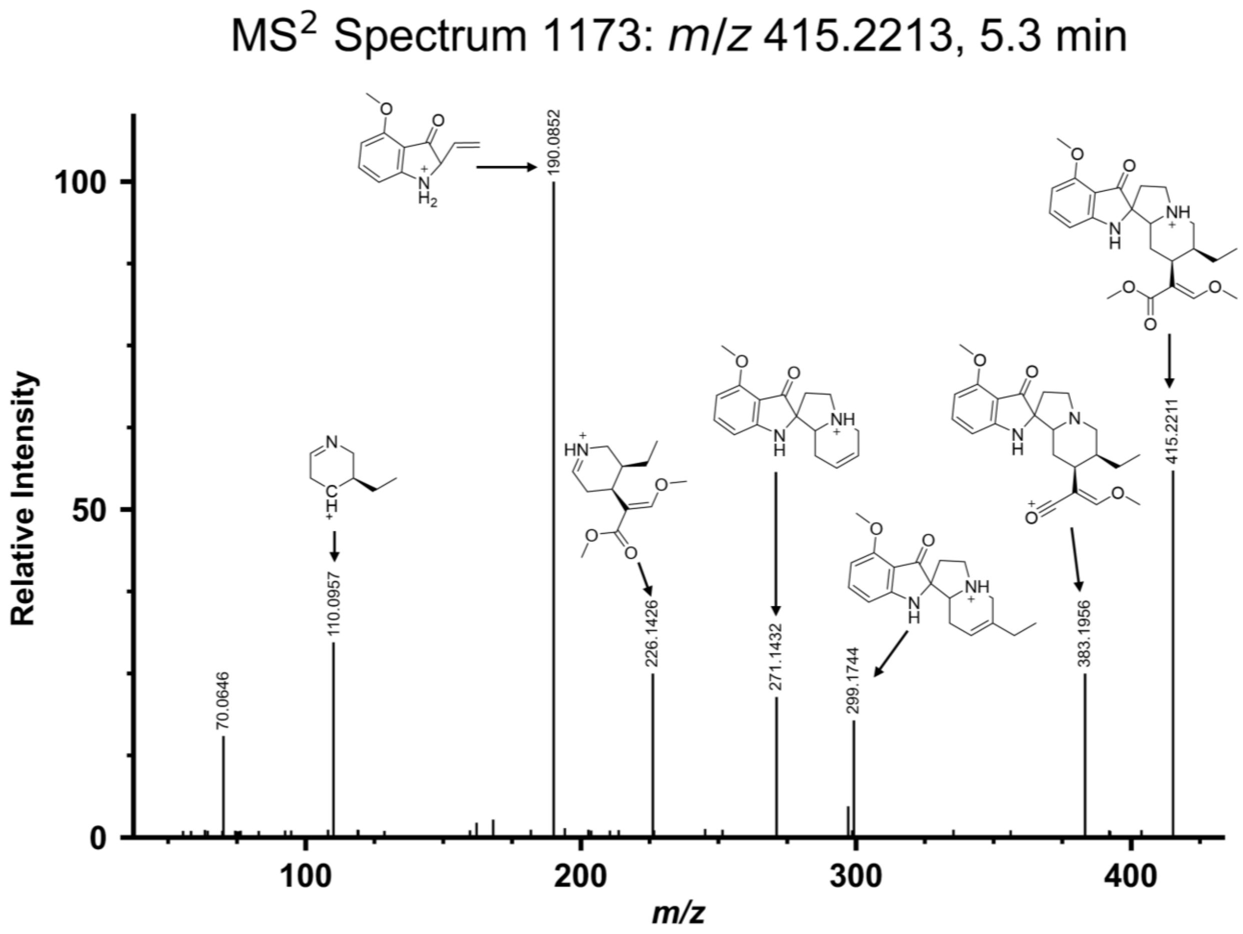

Figure S18: MS2 spectra of mitragynine metabolite linked to Table S2. The structure for the precursor metabolite adduct was annotated along with possible fragment structures.

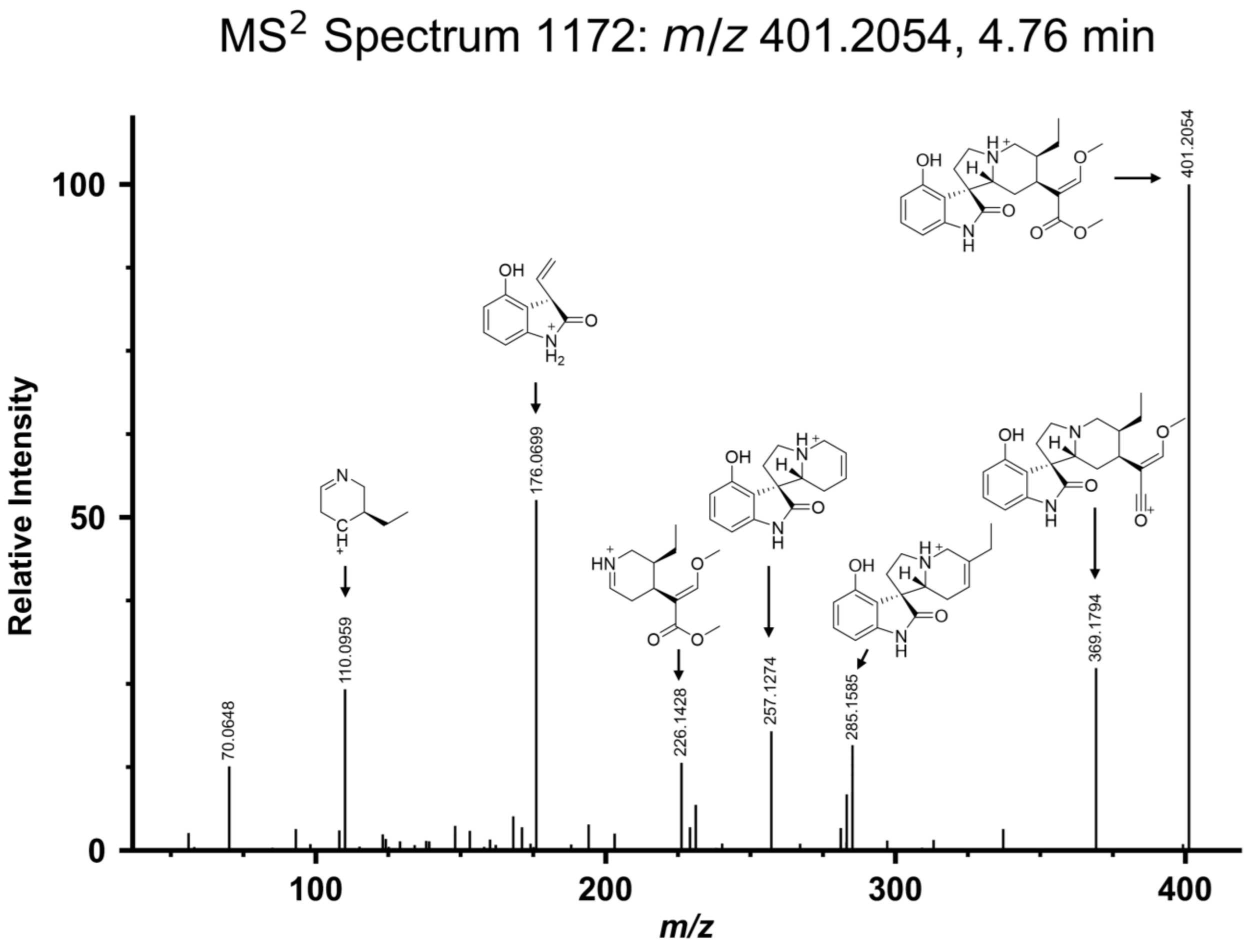

Figure S19: MS2 spectra of speciofoline linked to Table S2. The structure for the precursor adduct was annotated along with possible fragment structures.

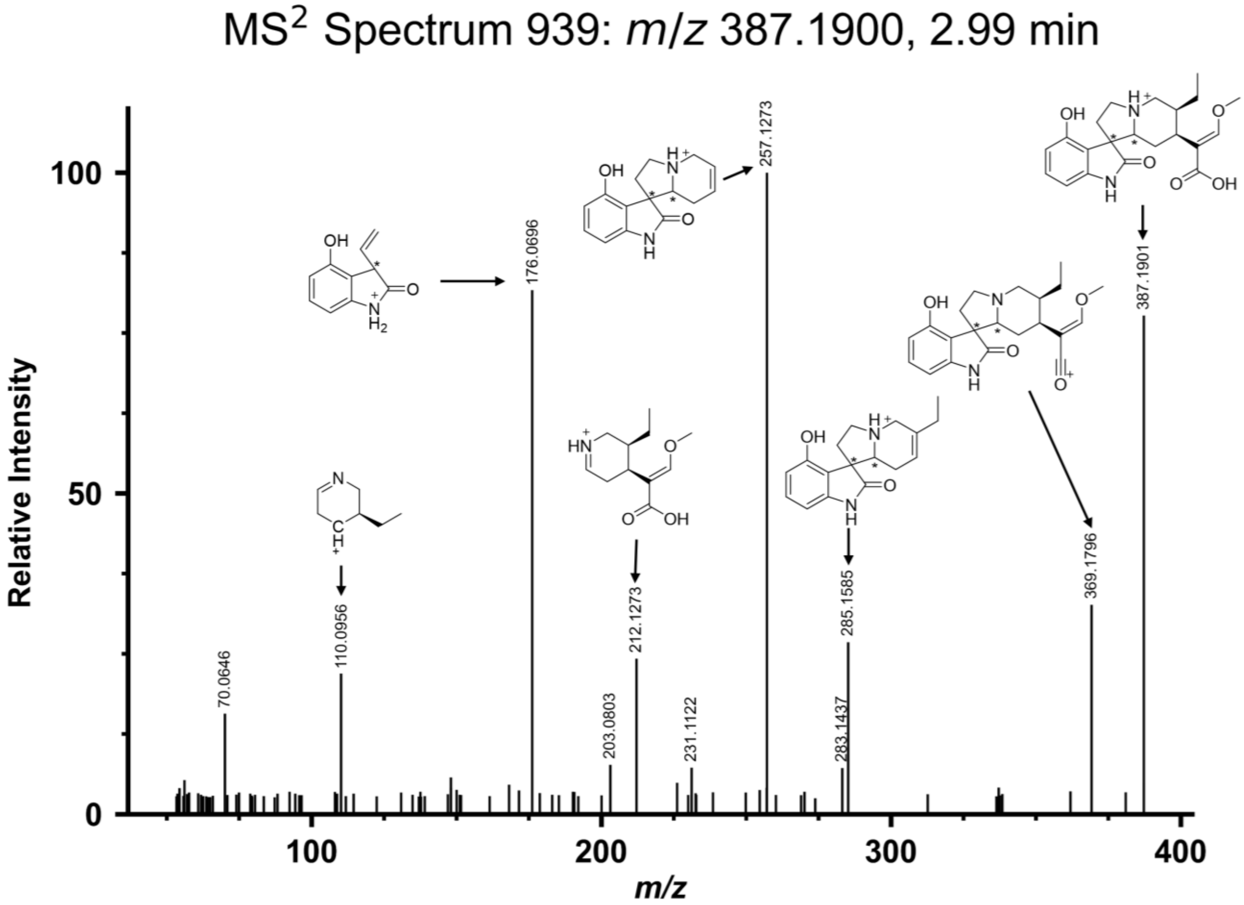

Figure S20: MS2 spectra of speciofoline metabolite linked to Table S2. The structure for the precursor metabolite adduct was annotated along with possible fragment structures. Asterisks indicate stereocenters where stereoisomerization can occur, where the stereochemistry cannot be hypothesized.

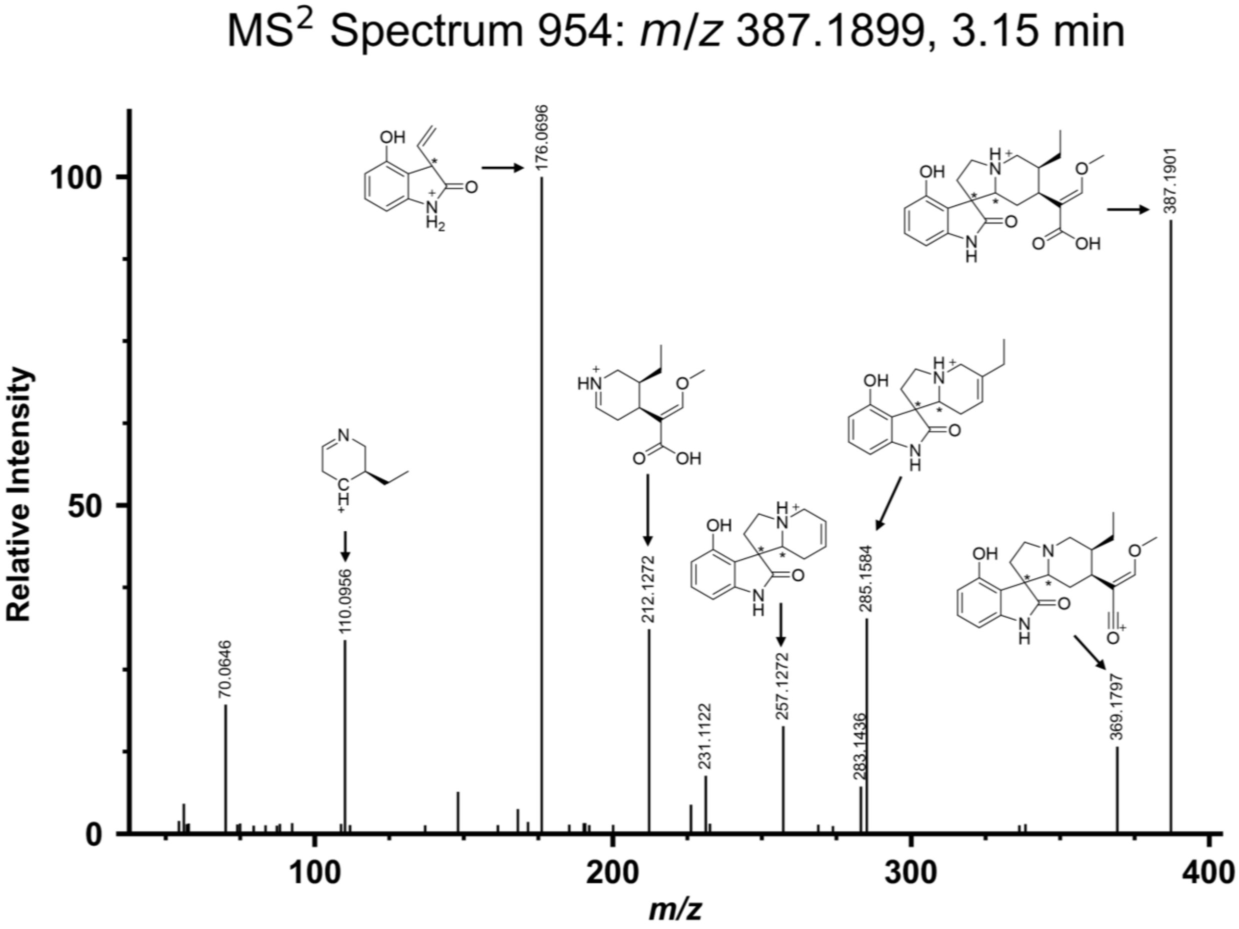

Figure S21: MS2 spectra of speciofoline metabolite linked to Table S2. The structure for the precursor adduct was annotated along with possible fragment structures. Asterisks indicate stereocenters where stereoisomerization can occur, where the stereochemistry cannot be hypothesized.

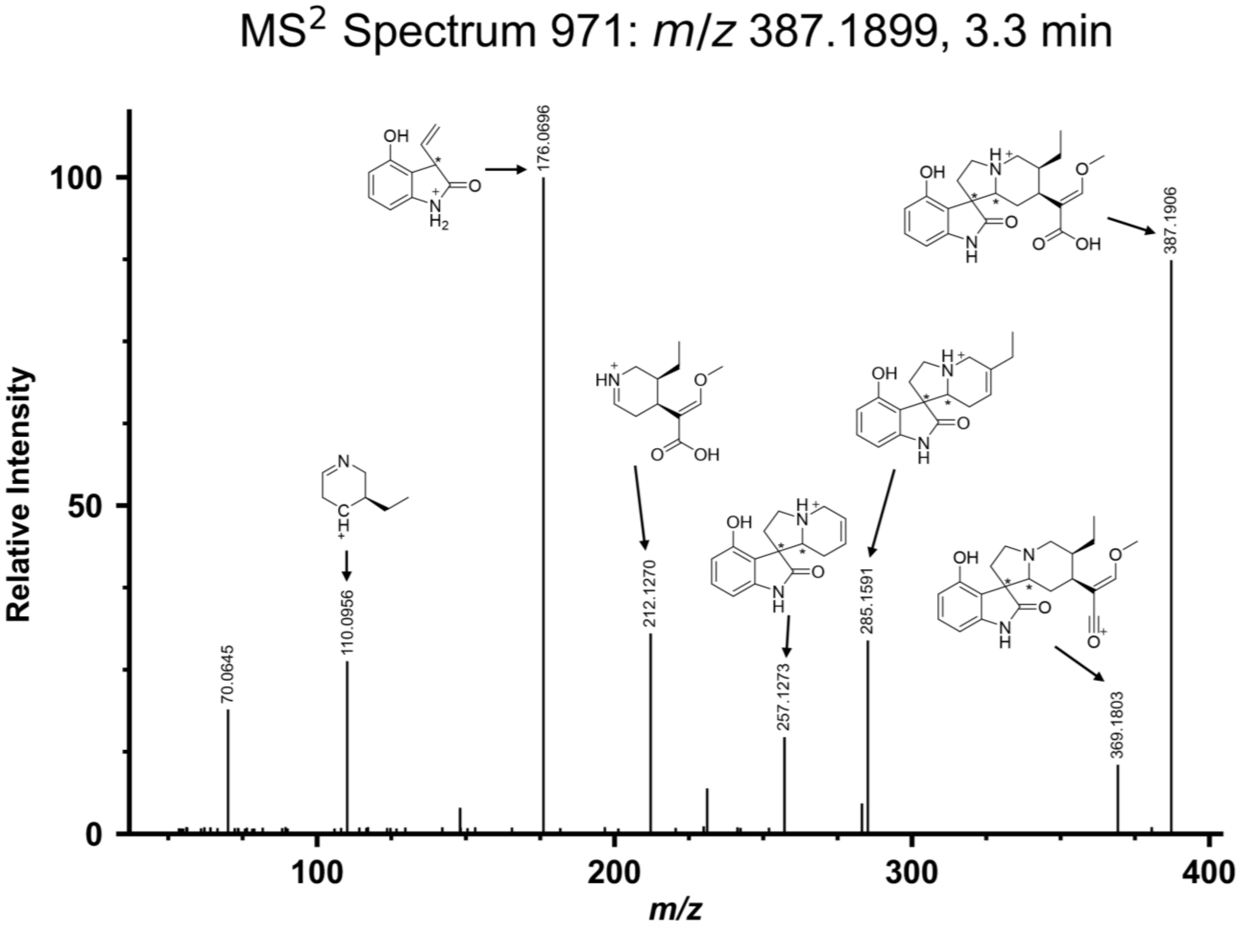

Figure S22: MS2 spectra of speciofoline metabolite linked to Table S2. The structure for the precursor adduct was annotated along with possible fragment structures. Asterisks indicate stereocenters where stereoisomerization can occur, where the stereochemistry cannot be hypothesized.

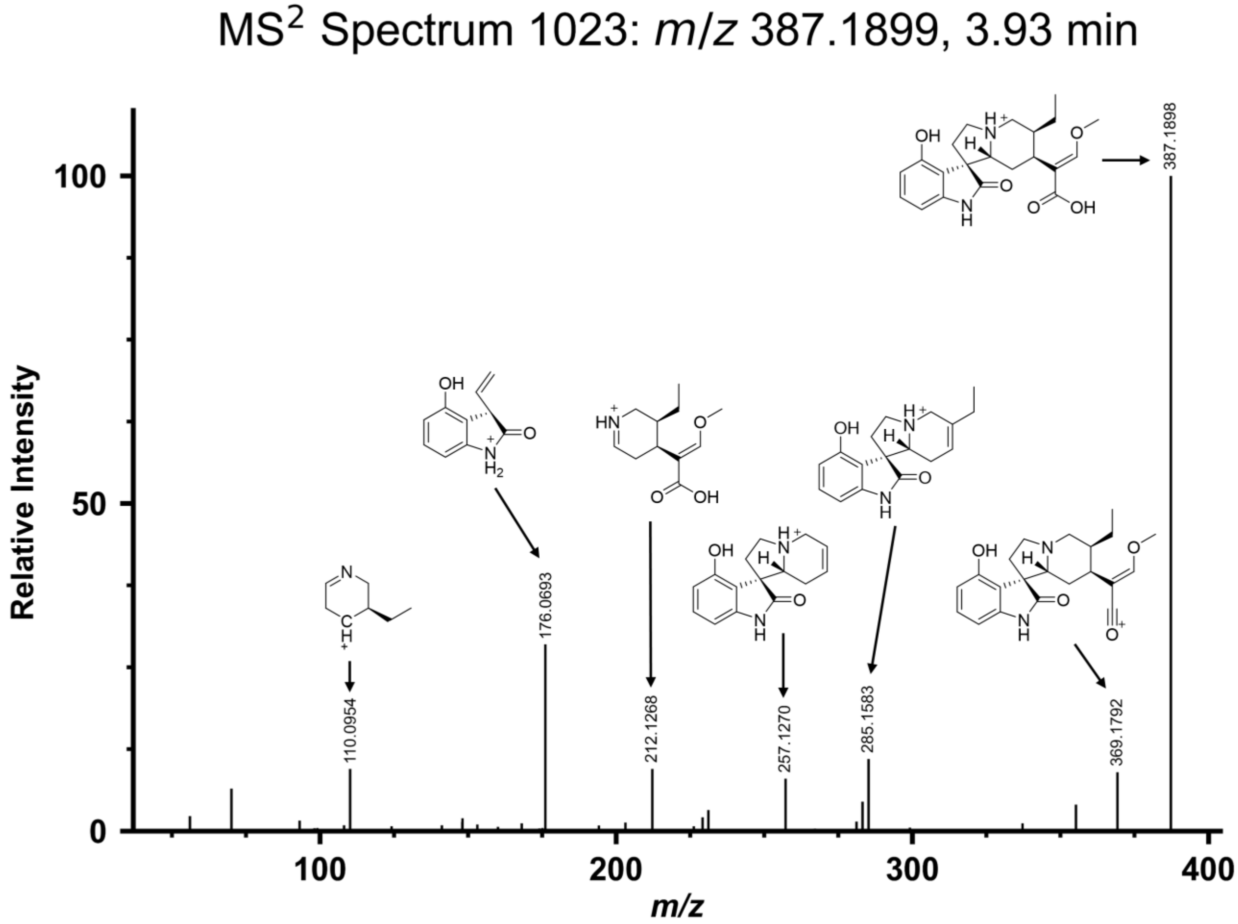

Figure S23: MS2 spectra of speciofoline metabolite linked to Table S2. The structure for the precursor adduct was annotated along with possible fragment structures.

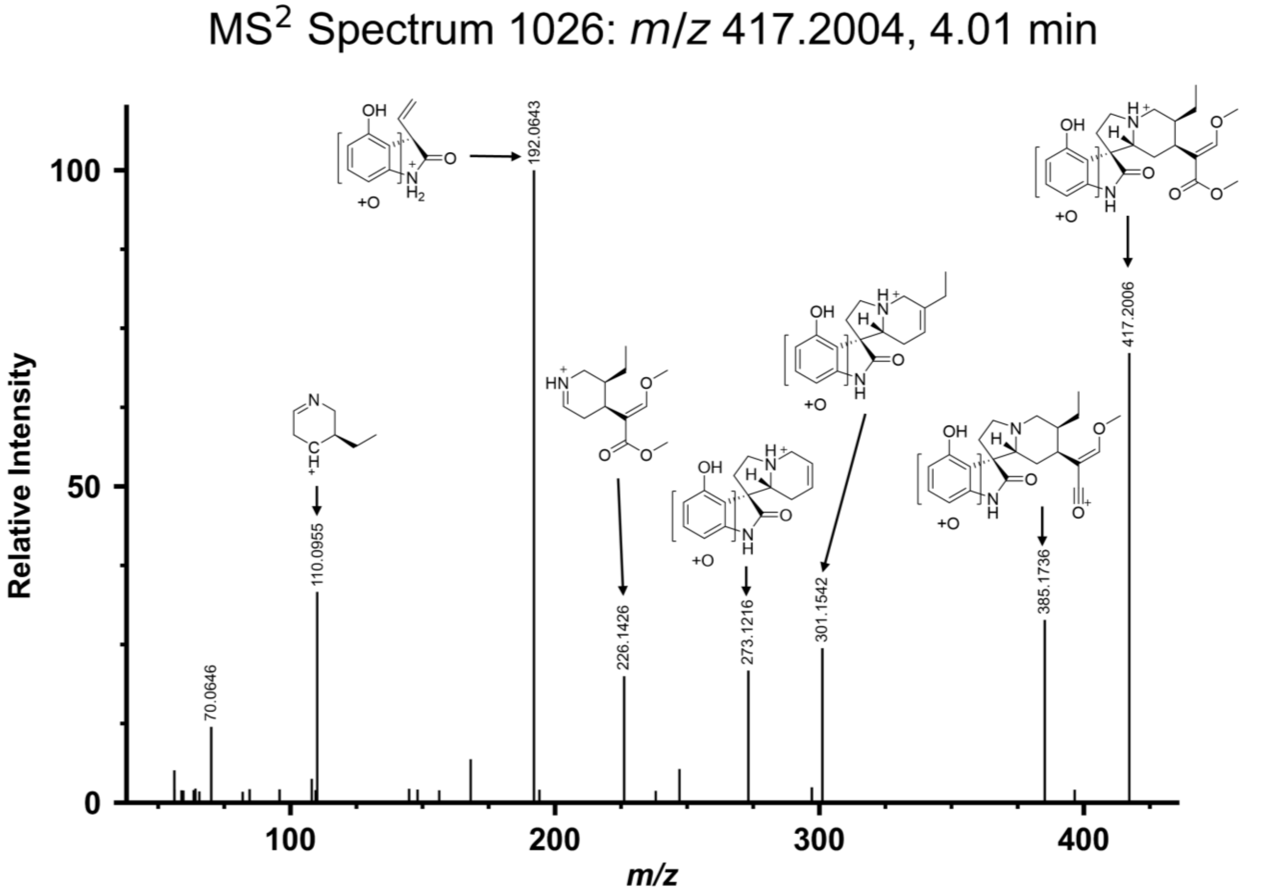

Figure S24: MS2 spectra of speciofoline metabolite linked to Table S2. The structure for the precursor adduct was annotated along with possible fragment structures.

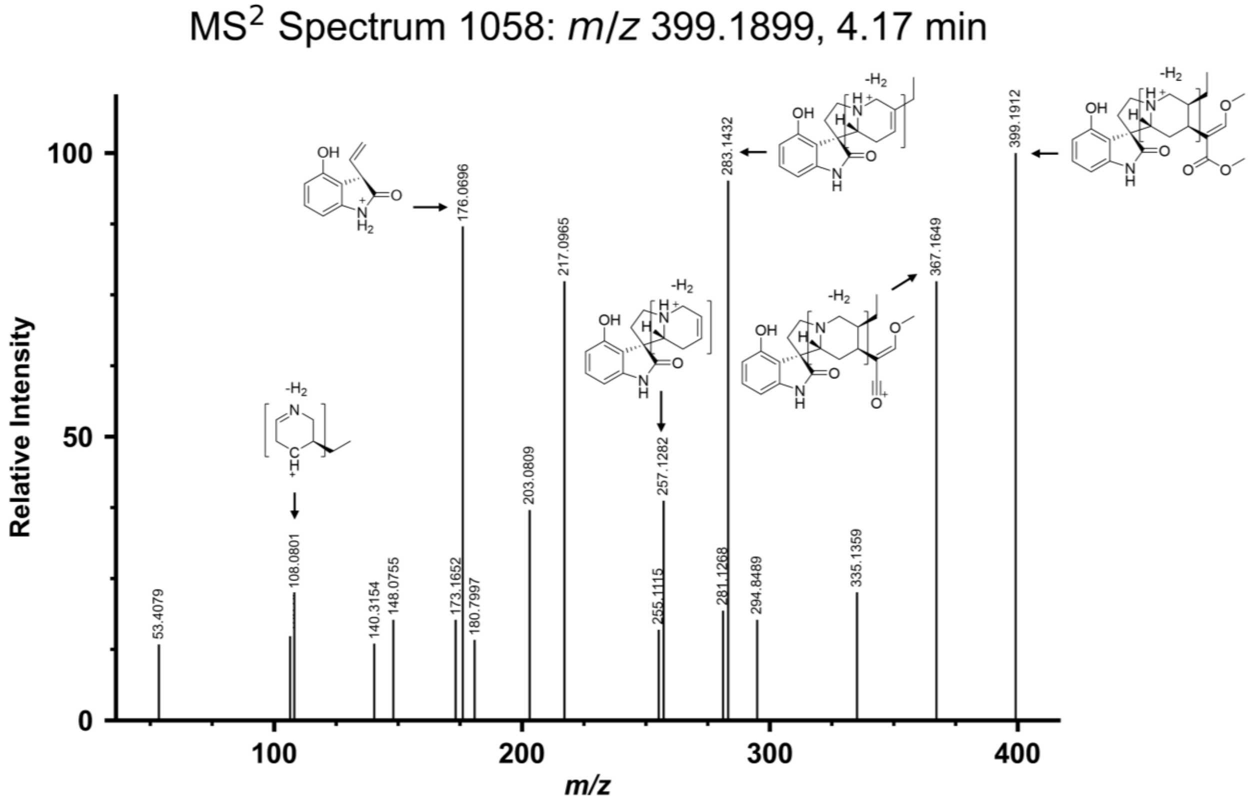

Figure S25: MS2 spectra of speciofoline metabolite linked to Table S2. The structure for the precursor adduct was annotated along with possible fragment structures.

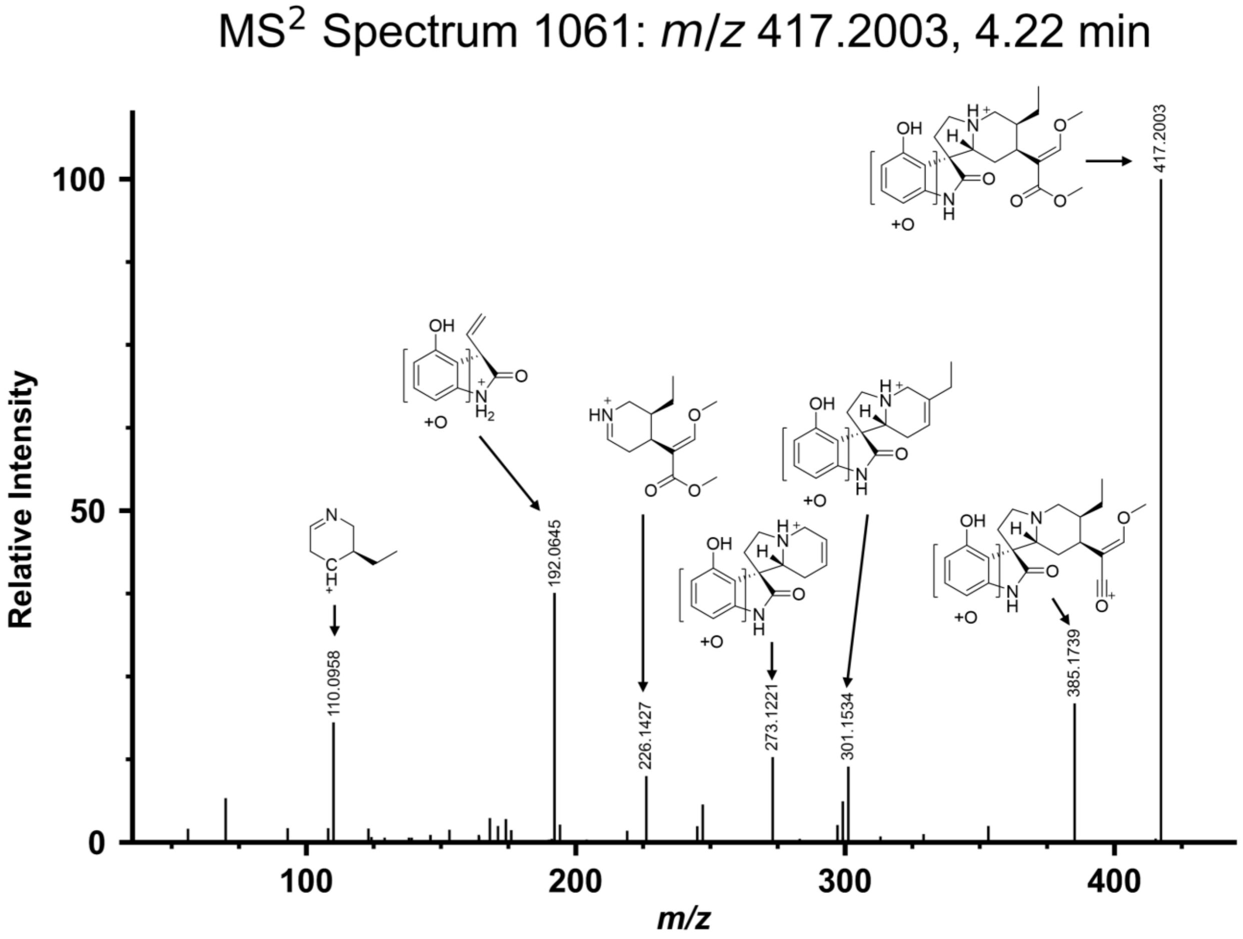

Figure S26: MS2 spectra of speciofoline metabolite linked to Table S2. The structure for the precursor adduct was annotated along with possible fragment structures.

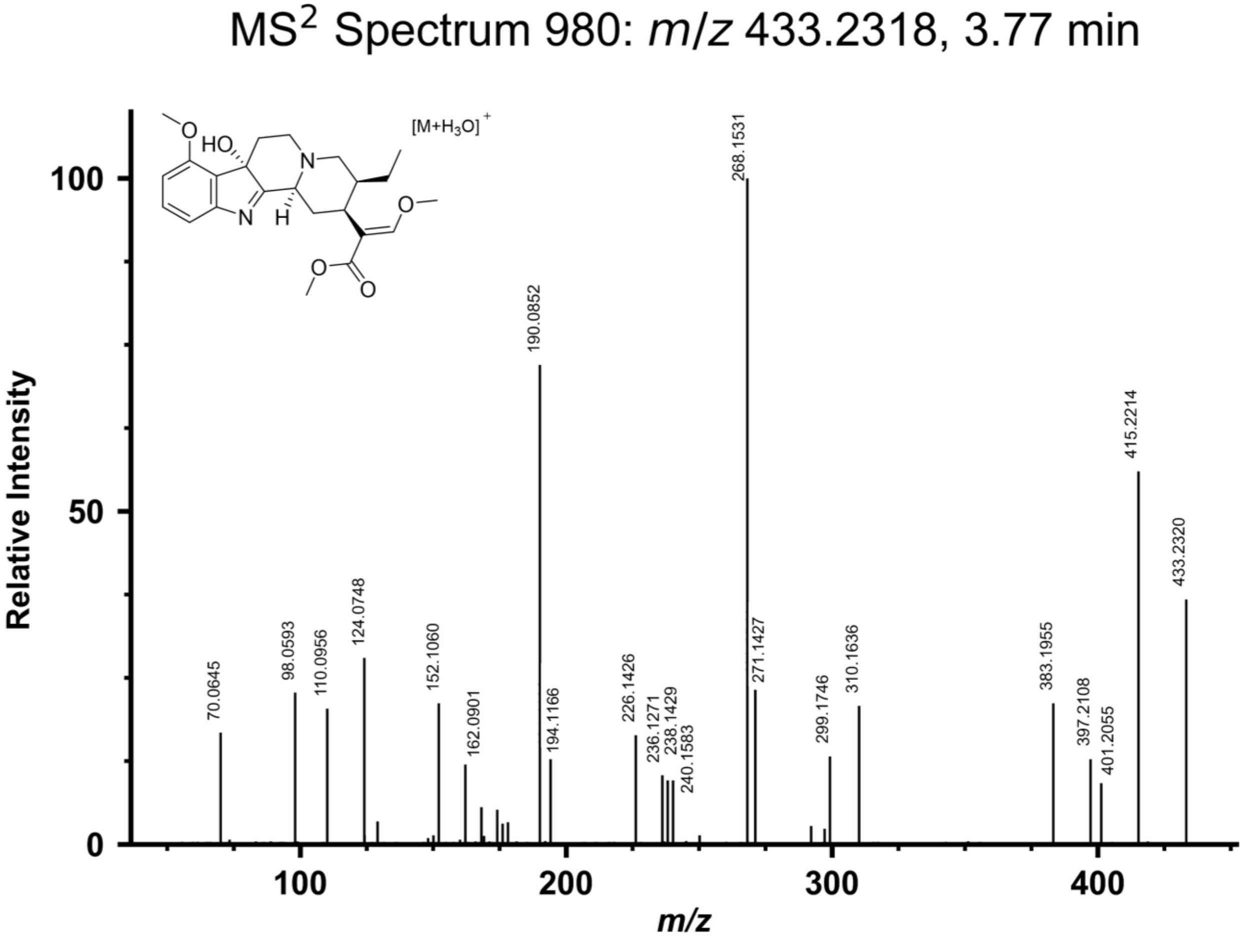

Figure S27: MS2 spectra of mitragynine metabolite linked to Table S2. This is proposed to be a water adduct of 7OH-mitragynine, due to its identical retention time and appearance of key fragments at m/z 190, 226, 238, 397, and 415. However, more work is needed to properly identify this metabolite as fragment m/z 271 is indicative of a spiro-pseudoindoxyl system (see Figure S18). This could be due to convoluted spectra from inadequate chromatographic separation.
